# The colonic epithelium plays an active role in promoting colitis by shaping the tissue cytokine profile

**DOI:** 10.1371/journal.pbio.2002417

**Published:** 2018-03-29

**Authors:** Jesse Lyons, Phaedra C. Ghazi, Alina Starchenko, Alessio Tovaglieri, Katherine R. Baldwin, Emily J. Poulin, Jessica J. Gierut, Casie Genetti, Vijay Yajnik, David T. Breault, Douglas A. Lauffenburger, Kevin M. Haigis

**Affiliations:** 1 Cancer Research Institute, Beth Israel Deaconess Cancer Center and Department of Medicine, Harvard Medical School, Boston, Massachusetts, United States of America; 2 Department of Biological Engineering, Massachusetts Institute of Technology, Cambridge, Massachusetts, United States of America; 3 Division of Endocrinology, Boston Children’s Hospital, Boston, Massachusetts, United States of America; 4 Department of Pediatric Gastroenterology, Massachusetts General Hospital, Boston, Massachusetts, United States of America; 5 Department of Medicine, Division of Gastroenterology, Massachusetts General Hospital, Boston, Massachusetts, United States of America; 6 Department of Pediatrics, Harvard Medical School, Boston, Massachusetts, United States of America; 7 Harvard Stem Cell Institute, Cambridge, Massachusetts, United States of America; 8 Harvard Digestive Disease Center, Boston, Massachusetts, United States of America; New York University, United States of America

## Abstract

Inflammatory bowel disease (IBD) is a chronic condition driven by loss of homeostasis between the mucosal immune system, the commensal gut microbiota, and the intestinal epithelium. Our goal is to understand how these components of the intestinal ecosystem cooperate to control homeostasis. By combining quantitative measures of epithelial hyperplasia and immune infiltration with multivariate analysis of inter- and intracellular signaling, we identified epithelial mammalian target of rapamycin (mTOR) signaling as a potential driver of inflammation in a mouse model of colitis. A kinetic analysis of mTOR inhibition revealed that the pathway regulates epithelial differentiation, which in turn controls the cytokine milieu of the colon. Consistent with our in vivo analysis, we found that cytokine expression of organoids grown ex vivo, in the absence of bacteria and immune cells, was dependent on differentiation state. Our study suggests that proper differentiation of epithelial cells is an important feature of colonic homeostasis because of its effect on the secretion of inflammatory cytokines.

## Author summary

Chronic inflammation of the gastrointestinal track is the common defect shared by inflammatory bowel diseases (IBDs), such as Crohn’s disease and ulcerative colitis, which affect many people around the world. However, the genetic and physiologic complexities of IBDs have made it difficult to identify therapeutically tractable drivers of disease that can alleviate the symptoms. We reasoned that this complexity is probably originated by a smaller number of dysregulated signaling pathways, and therefore, a “protein-centric” approach would be more suited to identify new therapeutic targets. To this end, in this study we profiled the expression and phosphorylation status of proteins that mediate signaling between and within cells in a mouse model of colitis. We found that hyperactivated mammalian target of rapamycin (mTOR) signaling interferes with the proper differentiation of epithelial cells, which promotes colitis by altering the epithelial inflammatory cytokine secretion in the colon.

## Introduction

IBD, comprised of Crohn’s disease (CD) and ulcerative colitis (UC), is characterized by chronic inflammation of the gastrointestinal tract. The symptoms of IBD, which include diarrhea, abdominal pain, and intestinal blockage, are chronic and debilitating and, in extreme cases, can result in death. Treatment options include steroids, aminosalicylates, and targeted therapies such as tumor necrosis factor alpha (TNF-α) neutralizing antibodies, but many patients become refractory to all of these therapies and require surgery [1]. As such, there is great need for medical therapies that exhibit a more durable response.

Generally, IBDs are understood to result from a loss of homeostasis between the intestinal epithelium, the mucosal immune system, and the gut microbiome. Genetic approaches, such as genome-wide association studies (GWAS), have identified numerous single nucleotide polymorphisms (SNPs) associated with IBD risk, many of which are involved in adaptive and innate immune function [2, 3]. While the immune system is clearly one of the key drivers of IBD, the intestinal epithelium also plays a central role in preventing inflammation by maintaining homeostasis of the gut. It represents a critical physical barrier between the commensal flora and the immune system that resides in the lamina propria and also plays a central role in antigen presentation [4, 5]. Without the epithelium, it would be impossible to maintain proper homeostatic control of the mucosal immune system. Indeed, IBD results from hyperactivation of the immune system in response to commensal or pathogenic bacteria in the context of epithelial damage.

Although experimental approaches indicate that changes to any of the 3 main components of the intestinal ecosystem can trigger IBD, the resulting clinical presentation of patients with CD or UC is indistinguishable, regardless of initiating event. Because of this, we have hypothesized that disparate initiating events converge on a shared, self-sustaining disease network composed of pathologic changes to all 3 main components of the gut ecosystem. Therefore, therapeutic approaches that target genetic initiating events might fail because inhibition of the triggering event would be ineffective in a self-sustaining disease state. An approach that focuses directly on these convergent downstream physiological processes and signaling pathways may identify novel targets that provide sustained therapeutic value for a larger number of patients.

In this paper, we have applied a protein-centric systems biology approach to characterize, at the tissue level, the key molecular and phenotypic features that comprise this convergent chronic inflammatory disease state. Through this work, we developed quantitative phenotypic readouts of inflammation and measured more than 50 inter- and intracellular signaling molecules that are associated with inflammation. Using dimensionality reduction algorithms, we predicted mammalian target of rapamycin (mTOR) signaling as a driver of colitis. While mTOR plays numerous roles that may be linked to IBD pathogenesis, including control of immune differentiation and activation and autophagy [6, 7], in this context we found that mTOR’s regulation of the differentiation state of the intestinal epithelium plays a key role in sustaining chronic inflammation. Using mouse models and in vitro 3D systems, we found that undifferentiated colonic epithelium produces high levels of innate immune cytokines and chemokines that drive inflammation, comparable to those proinflammatory molecules regulated by mTOR during colitis. Altogether, this work has resulted in a systems-scale model of colitis and has identified defective epithelial differentiation as a central mediator of chronic bowel inflammation. By extension, IBD patients, and particularly those with UC, who have hyperproliferative and undifferentiated colonic epithelium, may benefit from therapeutic approaches that induce differentiation to break the cycle of chronic inflammation.

## Results

### Systems-level characterization of murine colitis

To create a tissue-level systems model of chronic colitis, we utilized the T-cell transfer (TCT) model of colitis, which allowed us to control the timing and severity of inflammation in mice [8]. In this model, naïve T cells isolated from wild-type (WT) mice are injected into Rag1 null mice, which lack an adaptive immune system. In the absence of regulatory T cells (Tregs), the naïve T cells produce an inflammatory response to resident microflora, resulting in colitis with high penetrance (80%–90%) and a latency of 3–15 weeks. Our initial experimental population included 20 Rag1 null mice injected with naïve T cells and, as a negative control, 8 Rag1 null mice injected with Tregs (S11 Fig). Mice were weighed biweekly and assessed for symptoms of inflammation, such as diarrhea and rectal prolapse. After animals began to show severe weight loss and diarrhea, they were killed randomly over a range of time points to capture a spectrum of inflammatory states, an important feature for subsequent mathematical modeling. After humane killing, colons from individual mice were subdivided into matched sections for histology, immunophenotyping by flow cytometry, and multiplexed, Luminex-based protein measurement (S1 Fig).

**Fig 1:**
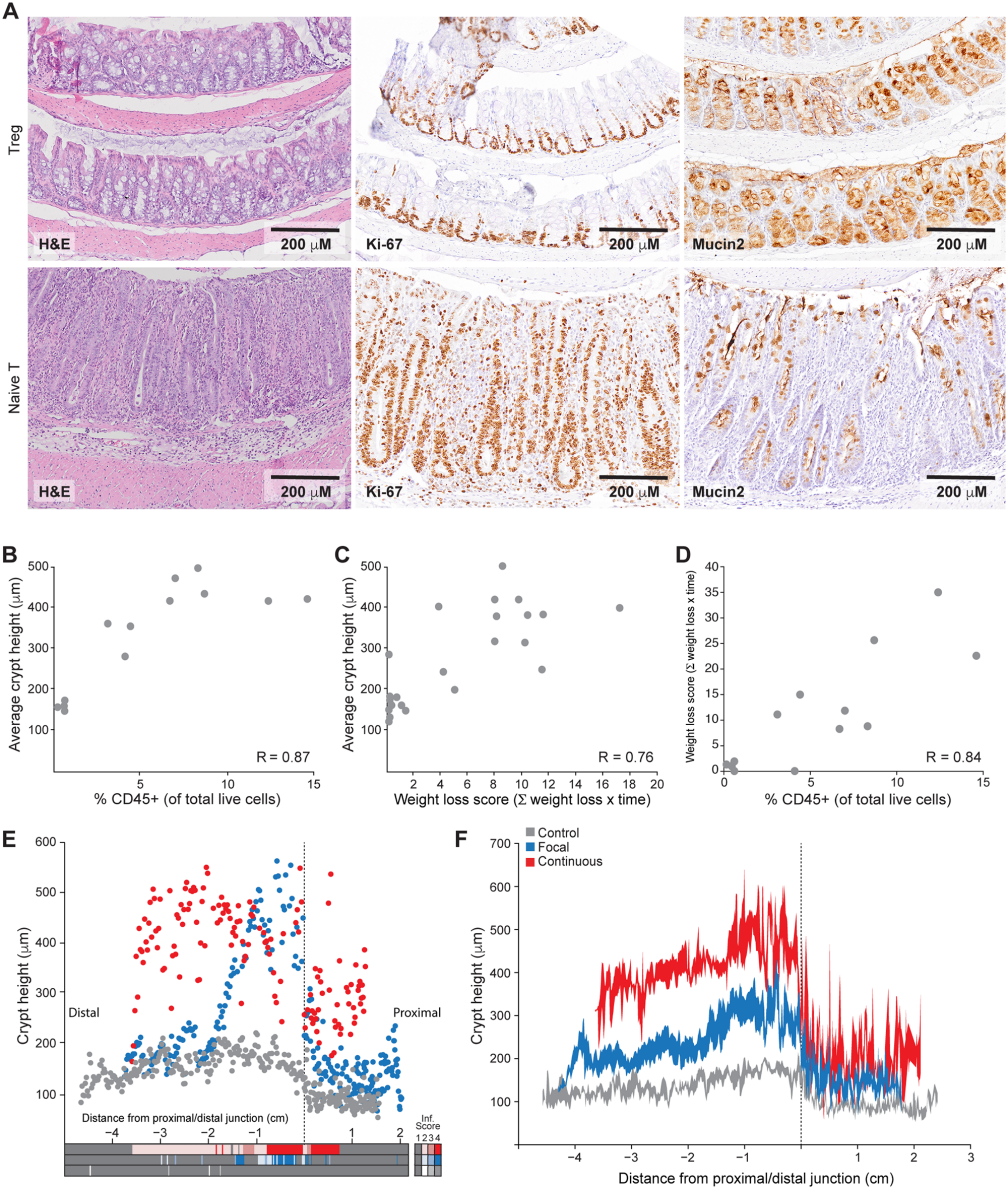
Phenotypic characterization of murine colitis. (A) Representative histology of colons from control regulatory T cell (Treg)-injected animals and naïve T-injected animals with inflammation. The left panels show hematoxylin–eosin (HE)-stained colons; the center panels show immunohistochemistry for Ki-67, a marker of undifferentiated cells; and the right panels show immunohistochemistry for mucin-2, a marker for goblet cells. (B) Plot of average crypt height in distal colon versus the percentage of total CD45+ immune cells in the tissue, showing a strong positive relationship. R was determined by Spearman correlation. (C) Plot of average crypt height in distal colon versus weight loss score, showing a strong linear relationship. R was determined by Pearson correlation. (D) Plot of the percentage of total CD45+ immune cells in the colon versus weight loss score, showing a strong linear relationship. R was determined by Pearson correlation. (E) Scatter plots of crypt height profiles for the animals from panel A. Each dot represents a single crypt measurement and its location within the colon from distal to proximal colon. Focal and continuous inflammation classes were defined based on these profiles, with focal inflammation showing the variable phenotype (blue dots) and continuous inflammation showing consistent distal colon hyperplasia (red dots). The box at the bottom provides the immune infiltrate score at each location where crypts were measured, showing co-occurrence of hyperplasia and foci of immune infiltration. (F) Average crypt height profiles for each group, with the standard error about the mean indicated in shaded regions at points binned in 500-μm increments. *N* = 6, 7, and 8 for control, focal inflammation, and continuous inflammation, respectively. Underlying numerical values for panels B–F are provided in S1 Data.

Naïve T-injected animals exhibited significant changes to the intestinal mucosa, including epithelial hyperplasia, expansion of the proliferative zone, loss of differentiation, and increased immune infiltrate (Fig 1A and S2 Fig). The majority (18/20) of the naïve T cell-injected animals showed some degree of crypt hyperplasia and immune cell infiltration, which were highly correlated to one another and with the amount of weight lost (Fig 1B–1D). The increased height of the colonic crypts was due to the dramatic increase in the number of epithelial cells per crypt (S2 Fig). Immunohistochemistry (S2 Fig) and immunophenotyping (S3 Fig) revealed that many immune cell types were present in increased numbers in the colons of animals with colitis. For example, in Rag1 null control animals, CD45+ cells made up only a small proportion of total colonic cells (0.1%–0.3%), and the majority of these cells were plasmacytoid dendritic cells (pDCs). In naïve T-injected animals, the percentage of CD45+ cells showed a strong correlation with crypt hyperplasia (Fig 1B), and this infiltrate was composed primarily of CD4+ T cells, macrophages, and neutrophils (S3 Fig), although the relative proportions of these cell types varied from animal to animal.

In the course of phenotyping animals with colitis, we noted that the histological manifestations of colitis were not uniform throughout the colon but rather restricted to specific colonic regions. In order to quantitatively measure inflammation as a function of the geographical position in the colon, we measured the thickness of the mucosa every 5 crypts along the entire length of the colon for every mouse. These measurements were used to generate scatter plots that graph crypt height as a function of longitudinal location along the colon (Fig 1E). In addition to crypt height measurements, an immune infiltration score was used to identify and locate areas of inflammation. Foci of infiltrating immune cells were rarely found in control animals but were found throughout the colons of naïve T-injected animals. These infiltrates were more prevalent in continuously inflamed colons and were closely associated with areas of crypt hyperplasia (Fig 1E). After analyzing our entire experimental cohort, we found that we could divide experimental animals into 2 discrete classes based on the extent of colitis (Fig 1F). Continuously inflamed animals exhibited significant crypt hyperplasia (300–600 μm/crypt) throughout the distal colon, while focally inflamed animals were defined by variable crypt hyperplasia beginning at the proximal/distal junction and spreading distally towards the rectum. While animals that were killed at later time points had a higher prevalence of colitis, the presence and severity of colitis were most closely associated with weight loss and the time since initial weight loss (Fig 1C and 1D). In follow-up experiments, by using this weight loss metric to select the timing of drug treatments, we were able to ensure that we treated only animals with established continuous inflammation. Together, these data establish the quantitative and qualitative histological features of chronic colitis, defining crypt hyperplasia as a tissue-level feature of colitis in this model and identifying expansion of the proliferative zone and loss of differentiation as key cellular features of disease.

Having established quantitative phenotypic measures of colitis, we sought to identify the inter- and intracellular signals underlying inflammation. Fresh tissue from the distal colon was lysed for Luminex-based analysis of 37 cytokines, chemokines, and growth factors and 17 phosphoproteins marking key signaling pathways (S4–S6 Figs). These 54 “signals” represented all of the commercially available Luminex-based assays for which we were able to detect signal above background in the mouse colon. Many of the signals were significantly different in control animals and those receiving adoptive transfer of CD45RBhi T cells, and these changes were not simply due to the adoptive transfer, since animals that received CD45RBhi T cells did not exhibit increased signaling prior to the onset of colitis (S7 Fig).

**Fig 2:**
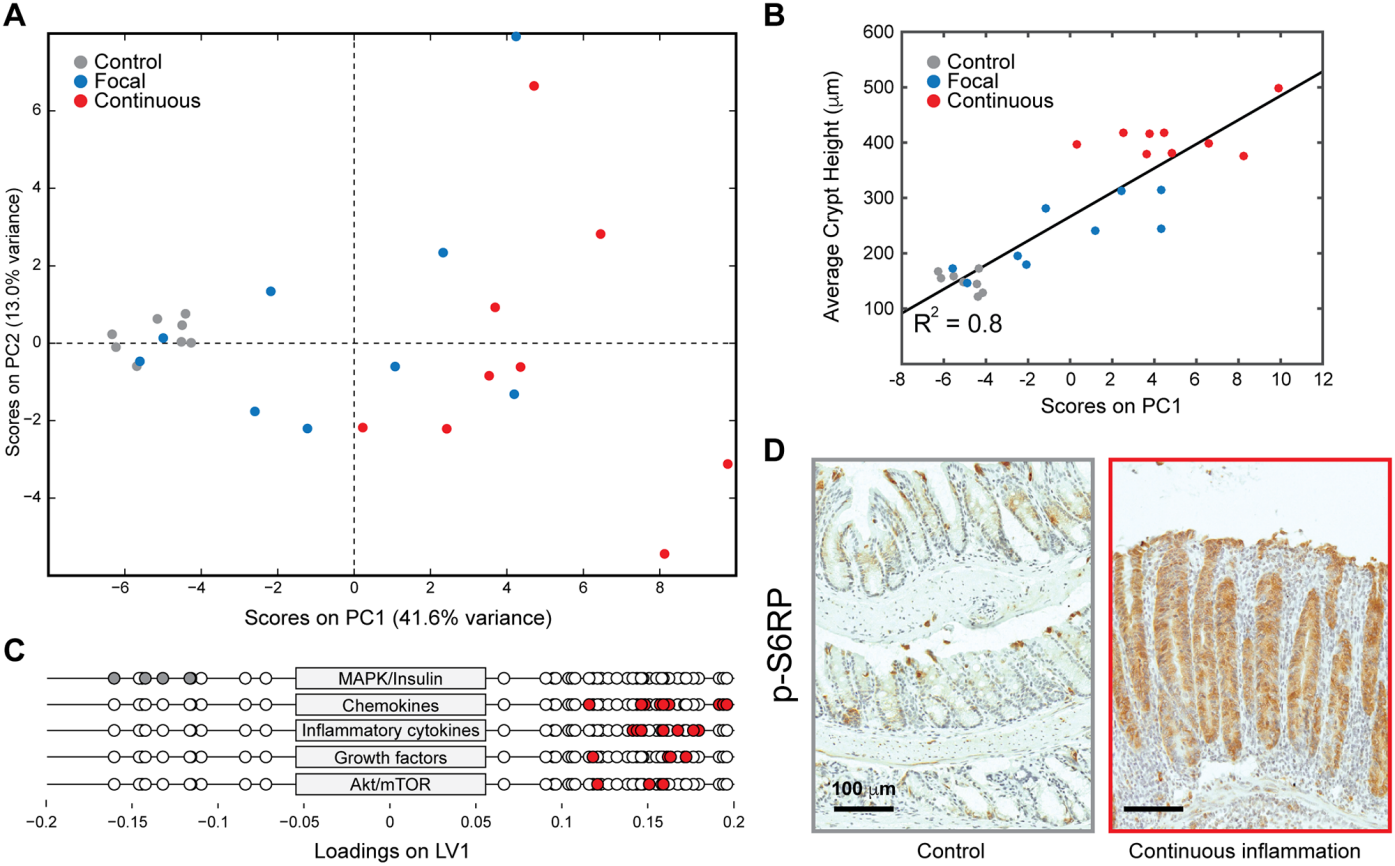
Multivariate molecular analysis of murine colitis. (A) Principal component analysis (PCA) based on Luminex measurements from distal colons identifies clusters of the control, focal inflammation, and continuous inflammation classes on principal component 1 (PC1). (B) Correlation between PCA and crypt height. For each animal, the score on PC1 was plotted against the average crypt height in the distal colon. This indicates that there is a strong correlation (R^2^ = 0.8) between PC1 and epithelial measures of inflammation. (C) Loadings on PC1 represented as single dots for each protein and grouped and color-coded by pathway or category. Signals on the right are positively correlated with inflammation. (D) Immunohistochemistry for p-S6 ribosomal protein (p-S6RP), a readout of the mammalian target of rapamycin (mTOR) pathway. p-S6RP was strongly induced in inflamed animals and was found primarily in epithelial cells. Underlying numerical values for panels A and B are provided in S1 Data. MAPK, mitogen-activated protein kinase.

We used principal component analysis (PCA) to cluster animals based on protein expression profiles and to identify key proteins driving this clustering. PCA showed a high degree of covariance between the proteins in the data set, and the majority of this variance was explained by the first principal component (PC1) (Fig 2A). Additionally, PC1 clustered each animal based on its degree of inflammation, with animals exhibiting continuous inflammation scoring positively on PC1, the focal inflammation animals scoring in the middle, and the control animals scoring negatively. Two of the naïve T cell-injected animals clustered with the negative control animals; however, these showed almost no hyperplasia or immune infiltration, representing the incomplete penetrance of this model (Fig 2A). Scores on PC1 were plotted against average crypt height and showed a strong linear correlation (R^2^ = 0.80), confirming that this principal component is strongly associated with inflammation (Fig 2B). PC1 showed strong positive loading for chemokines involved in macrophage, neutrophil, and T-cell chemotaxis (macrophage inflammatory proteins [MIPs], KC [keratinocyte chemoattractant], monocyte chemoattractant protein 1 [MCP-1], and RANTES [regulated on activation, normal T cell expressed and secreted]), inflammatory cytokines (interleukin [IL]-1s and IL-6), and growth factors (leukemia inhibitory factor [LIF] and vascular endothelial growth factor [VEGF]) (Fig 2C). T helper cell 2 (Th2) cytokines (IL-31 and IL-33) did not show positive loading, suggesting polarization of the T-cell response towards a Th1/Th17-type response, which is typical of CD and the TCT model of IBD [9]. Intracellular signaling molecules, such as Mek and the insulin receptor, showed strong negative loading, suggesting that these pathways are down-regulated in inflamed colons. The main intracellular signaling pathways up-regulated in inflamed animals included NF-κB and mTOR. We were particularly interested in the mTOR pathway because it was activated at multiple levels, including Akt, p70 S6K, and S6RP. The activation of mTOR in the context of colitis was not specific to the TCT model; we found that mTOR was activated in multiple genetic models of IBD (S8 Fig) and in a subset of human IBD patients (S9 Fig).

To determine whether mTOR signaling was generally up-regulated in the context of colitis or whether up-regulation in a specific cellular compartment accounted for the increased signal detected via Luminex, we performed immunohistochemistry for phosphorylated ribosomal protein S6 (p-S6) on colons from control and adoptive transfer mice. In control animals, we found high p-S6 signal only in intraepithelial lymphocytes, which is consistent with the well-characterized role for mTOR signaling in immune function (Fig 2D) [10]. In animals with colitis, by contrast, we detected strong p-S6 throughout the colonic epithelium (Fig 2D), suggesting that the increased mTOR signal that we detected derives primarily from ectopic activation in epithelial cells.

### mTOR activation suppresses colonic differentiation to promote inflammation

**Fig 3:**
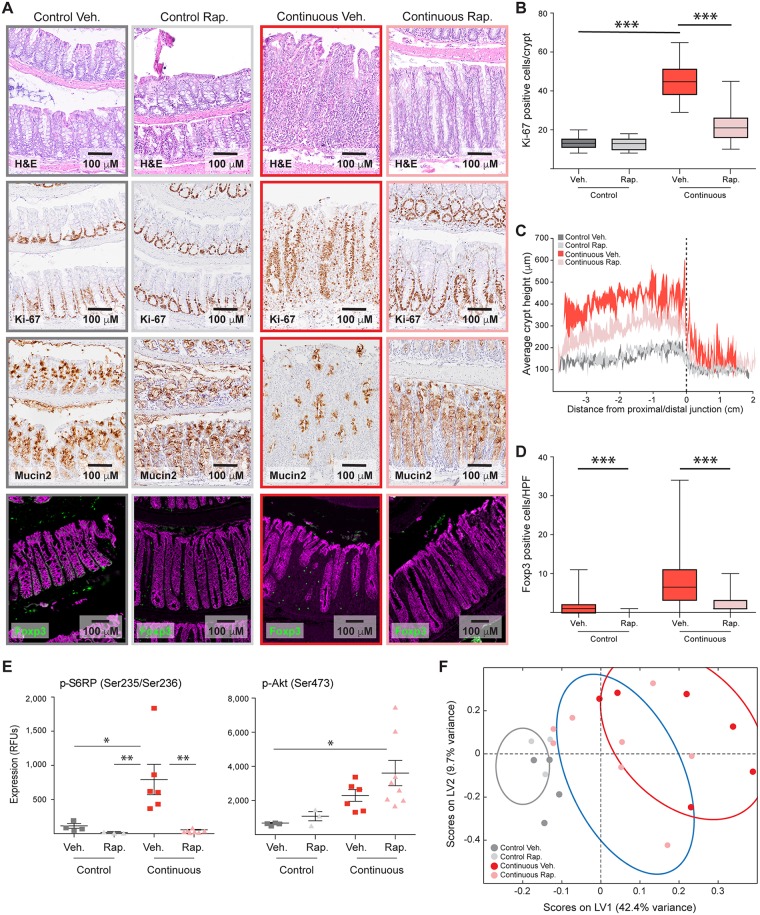
Mammalian target of rapamycin (mTOR) inhibition reduces inflammation. (A) Representative histology of colons from vehicle (Veh.)-treated control animals, rapamycin (Rap.)-treated control animals, vehicle-treated and naïve T-injected animals, and rapamycin-treated and naïve T-injected animals. Individual panels show (from top to bottom) hematoxylin–eosin (HE)-stained colons, Ki-67, mucin-2, and Foxp3, a marker of regulatory T cells (Tregs). (B) Measurement of proliferative zone heights via Ki-67 staining. There is a significant reduction in crypt height for inflamed rapamycin-treated animals, as determined by Mann-Whitney test. *** represents *p* ≤ 0.001. *N* = 100, 60, 80, and 120 for control + vehicle, control + rapamycin, naïve T + vehicle, and naïve T + rapamycin, respectively. (C) Average crypt height profiles for each treatment group, with the standard error about the mean indicated in shaded regions at points binned in 500-μm increments. *N* = 4, 3, 6, and 8 for control + vehicle, control + rapamycin, naïve T + vehicle, and naïve T + rapamycin, respectively. (D) Quantification of Foxp3-positive Tregs. There is a significant reduction in Tregs in the lamina propria of all animals treated with rapamycin. Tregs were quantified as the number of Foxp3-positive cells in a random high-powered field (HPF). *P*-values were determined by Mann-Whitney test. *** represents *p* ≤ 0.001. *N* = 75, 50, 100, and 75 for control + vehicle, control + rapamycin, naïve T + vehicle, and naïve T + rapamycin, respectively. (E) Luminex-based measurements of activating phosphorylation sites on multiple proteins in the mTOR pathway. Measurements are in arbitrary relative fluorescence units (RFUs). Horizontal and vertical lines represent mean +/− standard error. Significance was determined by one-way ANOVA with Tukey-Kramer post-test. * represents *p* ≤ 0.05; ** represents *p* ≤ 0.01. (F) Projection of signaling data from vehicle- and rapamycin-treated samples onto a partial least squares regression (PLSR) model built from untreated samples. Original samples used to generate the model project into the gray oval (control), blue oval (focal inflammation), and red oval (continuous inflammation). Underlying numerical values for panels B–F are provided in S1 Data.

In order to investigate a potential role for mTOR in driving colonic inflammation by affecting epithelial homeostasis, we tested the effects of the mTOR inhibitor rapamycin on colitis induced in the TCT model. To ensure experimental animals had severe inflammation, we utilized animals at late time points after adoptive transfer. We also used weight loss as a marker for onset of inflammation, beginning treatment on a mouse-by-mouse basis when an animal lost more than 1 gram of weight and did not regain the weight within 1 week. Inflamed animals were treated with rapamycin (5 mg/kg/day) for 2 weeks and then processed and analyzed as described above. Colons from rapamycin-treated animals exhibited a clear reduction in immune infiltrate, normalization of the proliferative zone at the bottom of the crypts, and restoration of goblet cell differentiation (Fig 3A and 3B). Quantification of crypt heights revealed a partial normalization of mucosal thickness and a significant decrease in average crypt height in rapamycin-treated animals (Fig 3C). Because rapamycin promotes expansion of Tregs in other systems [11], we explored whether the general reduction in inflammation following rapamycin treatment was associated with an increase in Tregs in the colonic lamina propria. While animals with colitis did have significant numbers of Tregs in the colon, those treated with rapamycin did not have greater numbers (Fig 3D)—in fact, they had fewer—indicating that rapamycin treatment did not suppress colitis in the TCT model by expanding the number of Tregs.

Analysis of phosphoproteins confirmed that signaling downstream of mTOR activity was strongly inhibited by rapamycin (Fig 3E), although phosphorylation of the upstream activator Akt appeared to be increased by rapamycin, presumably by relieving inhibition of the insulin-like growth factor receptor/insulin receptor substrate 1 (IRS1) axis [12]. We next sought to determine whether mTOR inhibition altered the local intracellular signaling network or whether it had a broad effect on the tissue-level inflammatory network (S4–S6 Figs). We first applied partial least squares regression (PLSR) to signaling data from our initial specimens in order to create a mathematical model linking dysregulated signaling to colitis, regressing to epithelial thickness. As with our original PCA model, the PLSR model was able to segregate animals with continuous inflammation from control animals and those with focal inflammation based on the accumulated signals that were measured in colonic lysates (Fig 3F). When the signaling data from animals treated with rapamycin were projected onto this PLSR model, 6 out of 8 rapamycin-treated animals clustered with the focally inflamed animals (Fig 3F). This reflects the fact that rapamycin treatment represses signaling directly downstream of mTOR and also the majority of the cytokines, chemokines, and growth factors associated with inflammation. Together, these results suggest that epithelial mTOR is a central regulator of intestinal inflammation in mouse models of IBD.

**Fig 4:**
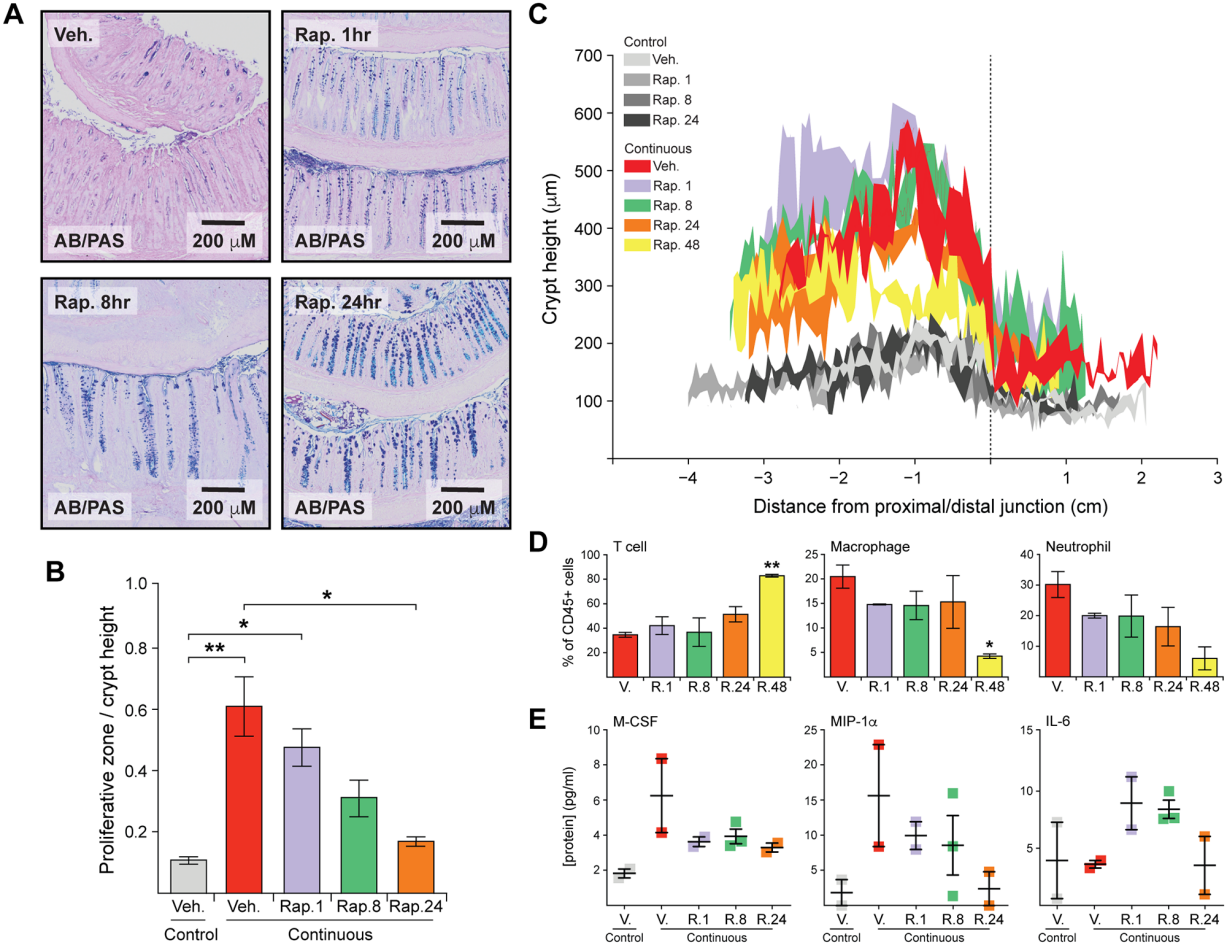
Temporal control of cellular and molecular inflammatory phenotypes by rapamycin. (A) Representative Alcian Blue/Periodic Acid Schiff (AB/PAS) stains for inflamed animals treated with vehicle (Veh.) or rapamycin (Rap.) for the indicated periods of time. Intracellular and extracellular mucins are stained dark purple and indicate the appearance of mature goblet cells. (B) Measurement of the proliferative zone before and after rapamycin. The proliferative zone is defined as the ratio of the highest phosphorylated histone H3 (PH3)-positive cell position in a crypt divided by the height of that crypt. For each animal, 12–25 crypts were measured, and bars represent the average proliferative zone for 2–3 animals. Significance was determined by one-way ANOVA with Tukey-Kramer post-test. * represents *p* ≤ 0.05; ** represents *p* ≤ 0.01. Bar graphs represent mean +/− standard error. (C) Average crypt height profiles for each treatment group, with the standard error about the mean indicated in shaded regions at points binned in 500-μm increments. *N* = at least 2 for each group. (D) Flow cytometric measurements of colonic immune infiltrate. Inflamed animals show an even mix of T cells, macrophages, and neutrophils, but rapamycin treatment leads to a loss of macrophages and neutrophils by 48 hours. Significance determined by ANOVA with Tukey-Kramer post-test. * represents *p* ≤ 0.05; ** represents *p* ≤ 0.01, both with respect to naïve T, vehicle control. *N* = 2–3 animals per condition. Bar graphs represent mean +/− standard error. (E) Representative cytokine expression patterns of response to acute rapamycin treatment. Macrophage colony-stimulating factor (M-CSF) drops immediately follow rapamycin exposure, while macrophage inflammatory protein 1-alpha (MIP-1α) declines slowly over 24 hours. Some proteins, like interleukin (IL)-6, transiently increase in expression following rapamycin treatment. *N* = 2–3 animals per condition. Bar graphs represent mean +/− standard error. Underlying numerical values for panels B–E are provided in S1 Data.

While long-term rapamycin treatment revealed a key role for mTOR in the maintenance of colitis, chronic inhibition does not provide mechanistic insight into the potential role that epithelial mTOR plays in this complex, tissue-level phenotype. In order to address this limitation, we treated inflamed animals acutely with rapamycin for 1, 4, 8, 24, or 48 hours. Within 1 hour of rapamycin treatment, goblet cells began to appear, and the proliferative zone began to normalize (Fig 4A and 4B). Within 24 hours, these cellular phenotypes were returned to nearly normal levels. By 48 hours, the inflammation-induced hyperplasia was reduced, reflecting the induced differentiation and decreased proliferation induced by mTOR inhibition (Fig 4C). Immune cell infiltrate changed more slowly following rapamycin treatment than did the epithelium, with a decrease in the proportion of macrophages and neutrophils only by 48 hours (Fig 4D). At that time point, the proportion of CD45+ cells that were T cells was significantly increased (Fig 4D). Cytokine and chemokine expression showed a variable kinetic response to rapamycin (S10 Fig). Some proteins decreased rapidly, within 1 hour of rapamycin treatment, to control levels, while others showed a graded decrease correlating to the goblet cell phenotype (Fig 4E). Some of the cytokines, like IL-6, even increased following rapamycin treatment (Fig 4E). These data are consistent with a kinetic model in which hyperactivated mTOR suppresses differentiation to promote colitis. Upon mTOR inhibition with rapamycin, colonic epithelial cells rapidly differentiate and cease to proliferate. Subsequently, the cytokine profile of the tissue changes, and, as a consequence, the complexion of the innate immune landscape returns to a state that resembles the normal colon.

### Rapamycin treatment does not affect the colonic microbiome

We next considered the possibility that rapamycin indirectly affected intestinal homeostasis by causing shifts in the composition of the commensal flora. To address this possibility, we profiled the fecal microbiome of control and inflamed animals, both before and after treatment with rapamycin (5 mg/kg/day for 2 weeks) (S11 Fig). Principal coordinate analysis (PCoA) of the beta diversity relationships between samples demonstrated that samples separated based on their inflammatory phenotype, but that rapamycin treatment did not shift the overall status of the microbiome, whether an animal had inflammation or not (S11 Fig). We also plotted the Bray-Curtis dissimilarity scores between all pairs of mice to see how individual mouse pre- and post-treatment scores compared with differences between animals. This analysis showed that the microbiome of control animals was most similar to other control animals, regardless of whether they received rapamycin (S11 Fig). The same was true for animals with inflammation. Although we did note that resolution of inflammation altered microbiome diversity and that the representation of certain bacteria, for example *Lactobacilli*, increased in inflamed mice after treatment, overall our data reveal that rapamycin itself does not alter microbial composition in this model.

### Induction of differentiation reduces inflammation

**Fig 5:**
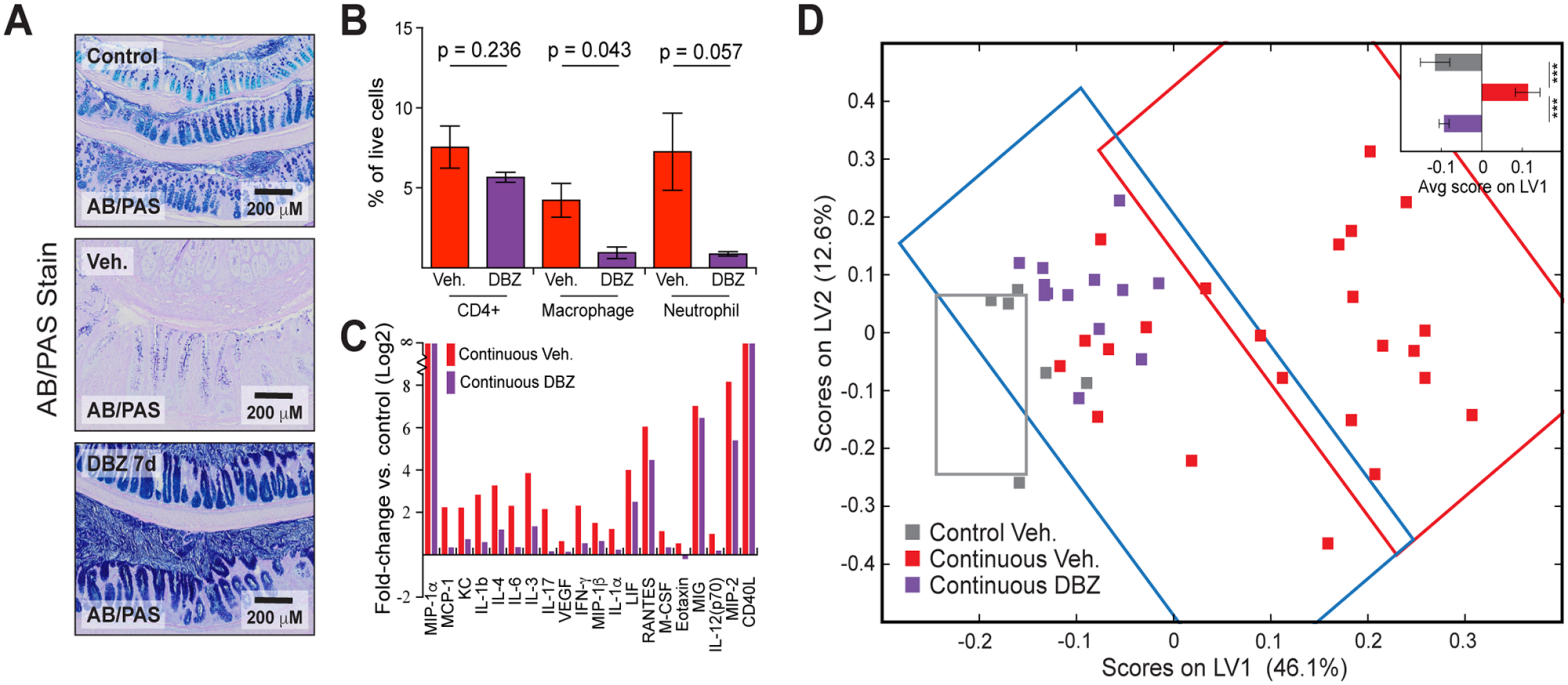
Goblet cell differentiation leads to reduced cytokine expression and innate immune cell infiltration. (A) Alcian Blue/Periodic Acid Schiff (AB/PAS) stain for mucin shows loss of goblet cells in inflamed animals that is restored by treatment with the Notch pathway inhibitor dibenzazepine (DBZ). (B) Immune infiltrate, as measured by flow cytometry expressed as a percentage of total cells, shows a reduction in macrophages and neutrophils following 1-week treatment with DBZ. Significance determined by unpaired *t* test, with *p*-values indicated above each cell type. *N* = 2–3 animals per condition. Bar graphs represent mean +/− standard error. (C) Inflammation-associated cytokine and chemokine expression was measured by Luminex and displayed as log2-fold changes versus control animals. These proteins represent the top 20 loadings from the partial least squares regression (PLSR) model described in Fig 5D. Data for individual animals are presented in S8 Fig. (D) DBZ- and vehicle (Veh.)-treated cytokine and chemokine measurements were projected onto a PLSR model built from non-drug-treated animals. DBZ-treated animals cluster with focal inflamed and noninflamed animals. Original samples used to generate the model project into the gray rectangle (control), blue rectangle (focal inflammation), and red rectangle (continuous inflammation). Underlying numerical values for panels B–D are provided in S1 Data. Avg, average; IFNγ, interferon gamma; IL, interleukin; LIF, leukemia inhibitory factor; LV1, latent variable 1; MCP-1, monocyte chemoattractant protein 1; MIG, mitokine induced by gamma interferon; MIP-1α/β, macrophage inflammatory protein 1-alpha/beta; M-CSF, macrophage colony-stimulating factor; RANTES, regulated on activation, normal T cell expressed and secreted; VEGF, vascular endothelial growth factor.

In our initial hypothesis-generating experiment and in the subsequent perturbation experiments, we found an inverse correlation between epithelial differentiation and colonic inflammation. This observation led us to ask whether induction of differentiation independently of mTOR inhibition would have a similar effect on tissue homeostasis. To this end, we used the gamma-secretase inhibitor dibenzazepine (DBZ), which induces goblet cell differentiation by inhibiting Notch signaling [13]. Animals with colitis were treated with 10 μmol/kg DBZ or vehicle by daily intraperitoneal (IP) injection for 1 week. DBZ-treated animals exhibited complete conversion of the colonic epithelium to goblet cells, with high mucous levels in almost every cell in the crypt (Fig 5A). Consistent with rapamycin treatment experiments, induction of differentiation via Notch inhibition was accompanied by a reduction in macrophages and neutrophils (Fig 5B). Interestingly, there was no change in the proportion of T cells within the colon, suggesting that their recruitment and maintenance within the tissue is not affected by systemic inhibition of Notch. This result is consistent with our prior observation that rapamycin treatment rapidly induced differentiation prior to having any effect on the number of T cells in the colon (Fig 4D). The rescue of differentiation was also associated with a decrease in the inflammation-associated chemokine profile (Fig 5C and S12 and S13 Figs), particularly those associated with innate immune cell chemotaxis. When the DBZ- and vehicle-treated samples were projected onto a cytokine-specific PLSR model built from the baseline samples, DBZ-treated samples clustered with focally inflamed and negative control animals, just like the rapamycin-treated animals in our previous experiment (Fig 5D). These experiments demonstrate that 2 mechanistically independent methods of inducing differentiation in the colonic epithelium lead to a reduction in the expression of inflammatory cytokines and chemokines and subsequent reduction in inflammatory immune infiltrate. These observations provide strong evidence that the epithelium is a key player, beyond its role as a barrier, in the inflammatory process and that this connection is tightly linked to differentiation state.

### Enrichment of transit-amplifying cells in inflamed epithelium

**Fig 6:**
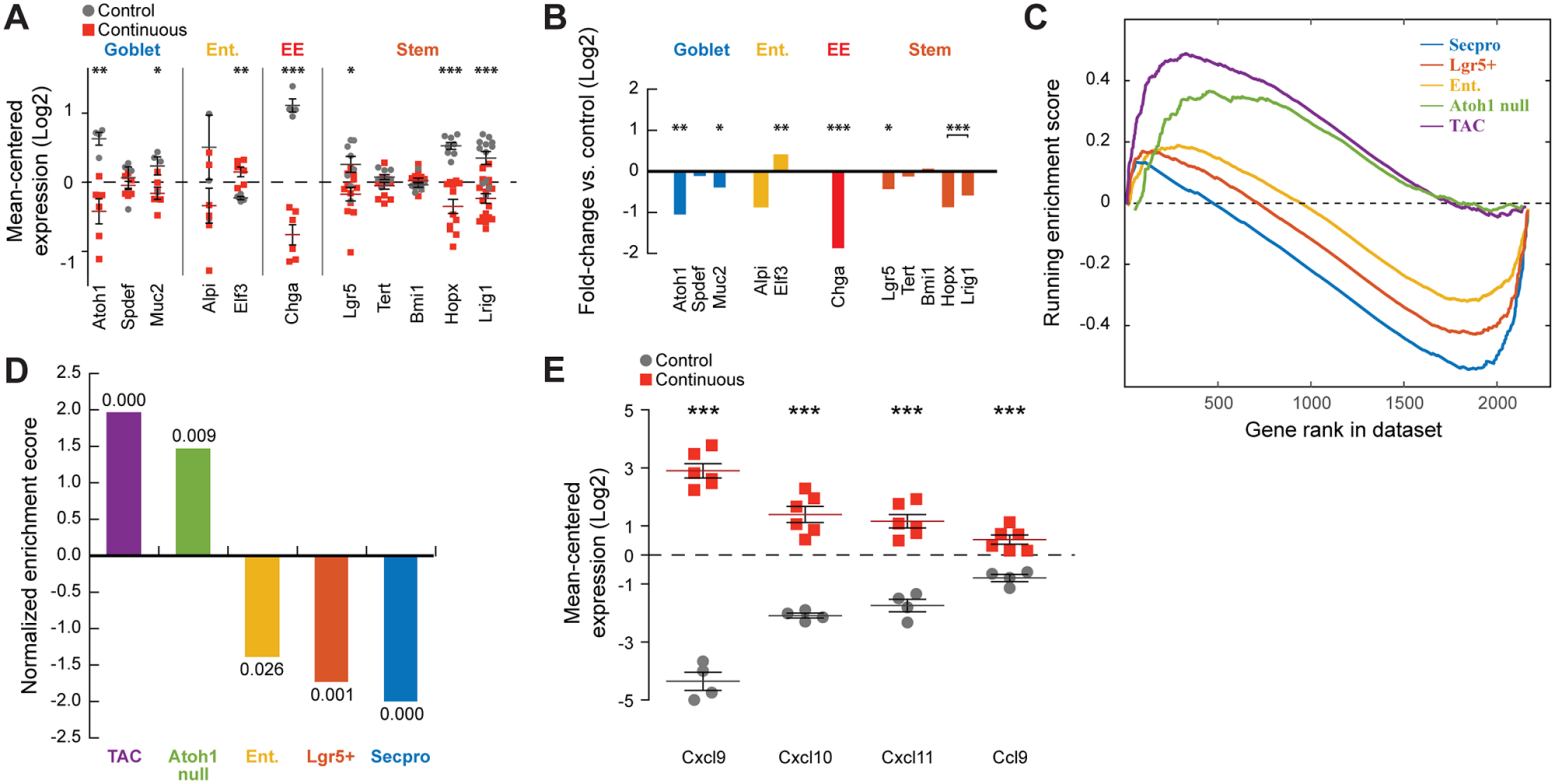
Expansion of transit amplifying cells in animals with colitis. (A) Markers of goblet cells, enterocytes (Ent.), enteroendocrine cells (EE), and stem cells measured by RNA microarray. Each gene was mean centered, and the means of inflamed and noninflamed groups are indicated in red and gray, respectively. Horizontal and vertical lines represent mean +/− standard error. Significance was determined by unpaired *t* test. * represents *p* ≤ 0.05, ** represents *p* ≤ 0.01, *** represents *p* ≤ 0.001. (B) Average fold change between inflamed and noninflamed groups for each of the genes in panel A. (C) Enrichment plots for experimentally derived gene sets for secretory progenitors (Secpro), stem cells, enterocytes (Ent), goblet cell-deficient epithelium (Atoh null), and transit-amplifying cell (TAC) epithelium. (D) Normalized enrichment scores for the gene sets indicated in panel C; false discovery rate (FDR) values are indicated above or below each bar. (E) Mean-centered expression values of the 4 chemokines found in the TAC gene set. The means of inflamed and noninflamed groups are indicated in red and gray, respectively. Horizontal and vertical lines represent mean +/− standard error. All genes showed a *p* < 0.001 in a *t* test, as indicated by ***. Underlying numerical values for panels A and C–E are provided in S1 Data.

Our studies indicate a clear role for the differentiation state of the epithelium in mediating cytokine and chemokine expression and innate immune recruitment in murine colitis. Nevertheless, the stage at which the epithelial cells were arrested remained unclear. In order to address this, we performed microarray analysis on 4 control and 6 inflamed colons (GEO accession GSE87317). Analysis of individual markers for different cell types suggested that there was a pronounced decrease of goblet cells and enteroendocrine cells and a moderate decrease of enterocytes and stem cells (Fig 6A and 6B), cellular phenotypes that we have confirmed by histologic analysis (Fig 1A and S2 Fig). Because transit amplifying cells (TACs) are defined functionally, the existing monovariate markers are not sufficient to assess their enrichment. In order to determine whether there was a relative increase in TACs in inflamed colon, we employed Gene Set Enrichment Analysis (GSEA) using published gene sets derived from epithelium enriched for various cell types. A stem cell gene set was derived from sorted Lgr5+ epithelial cells [14], while enterocyte and secretory progenitor gene sets were derived from various pharmacologic and genetic treatments [15]. Finally, a TAC signature was derived from regenerative epithelium composed of 97% TACs [16]. Using GSEA, we found de-enrichment for gene sets associated with secretory cells, enterocytes, and stem cells (Fig 6C and 6D). Conversely, we saw enrichment for genes associated with Atoh1 knockout (which lacks goblet cells) and the TAC signature. Importantly, several of the genes found in the TAC signature are chemokines, all of which were strongly induced transcriptionally in our microarray data set (Fig 6E). By contrast, there were no cytokines or chemokines present in any of the gene sets for stem or differentiated cell types. This observation confirms that cytokine expression is present within the epithelium itself and that this is enhanced in TACs, but not in stem cells or differentiated cells. Together, these analyses suggest that the suppressed differentiation and enhanced proliferation that is seen in colitis is the result of expansion of the TAC compartment and not the result of stem cell expansion.

### Differentiation state of the epithelium directly controls cytokine expression

**Fig 7:**
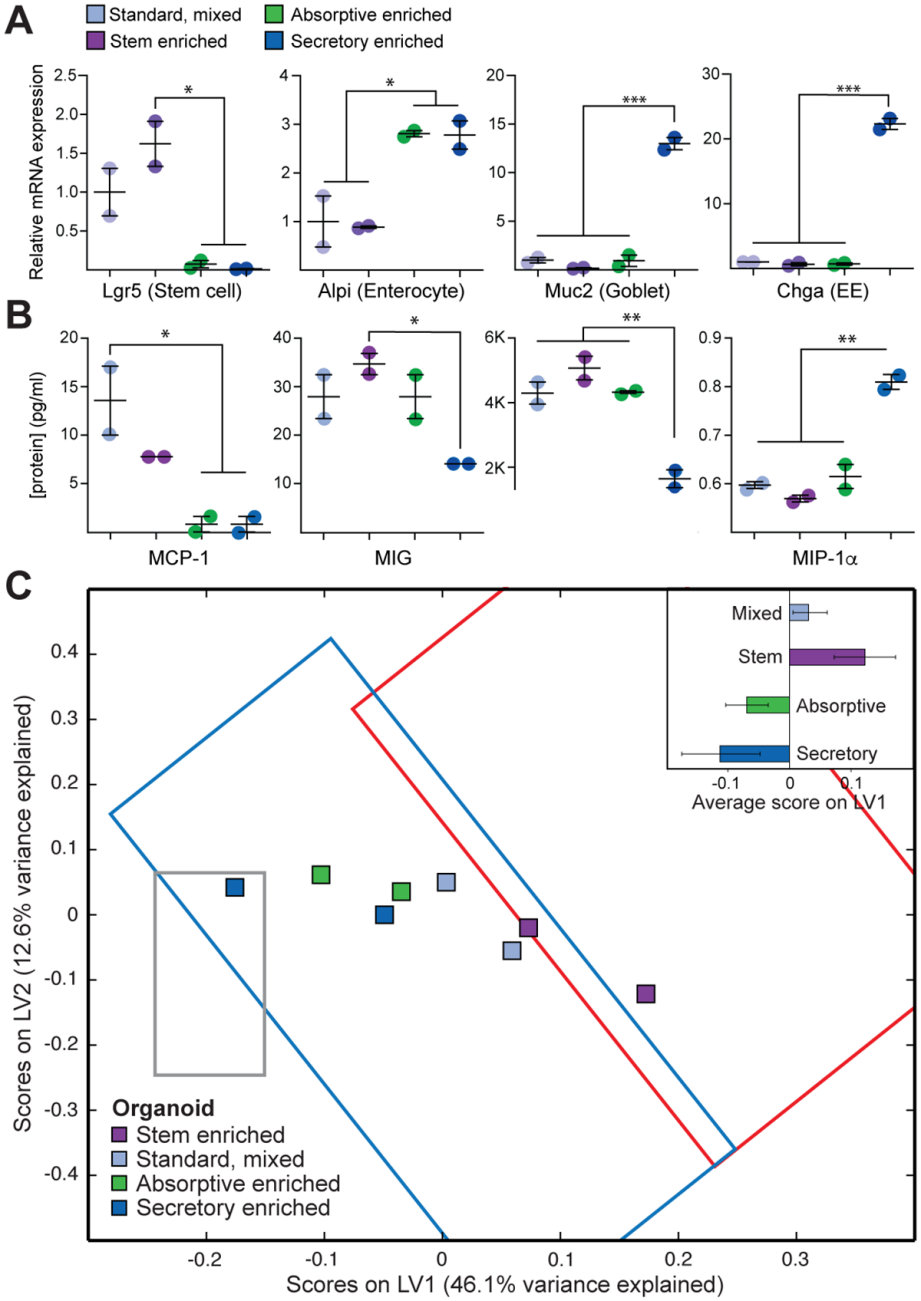
Epithelial differentiation reduces inflammatory cytokine expression in colonic organoids. (A) RNA markers of stem cells (*Lgr5*), enterocytes (*Alpi*), and goblet cells (*Muc2*) and enteroendocrine cells *(Chga*) were measured by quantitative polymerase chain reaction (qPCR) and normalized to standard culture condition organoid expression levels. Conditions for enrichment of stem cells or specific differentiated lineages can be found in the Materials and methods section. Horizontal and vertical lines represent mean +/− standard error. Significance was determined by ANOVA with Tukey-Kramer post-test. * represents *p* ≤ 0.05; *** represents *p* ≤ 0.001. (B) Representative cytokines, chemokines, and growth factors were measured by Luminex and represented in pg/ml. Horizontal and vertical lines represent mean +/− standard error. Significance was determined by ANOVA with Tukey-Kramer post-test. * represents *p* ≤ 0.05; ** represents *p* ≤ 0.01. (C) A partial least squares regression (PLSR) model built on cytokine, chemokine, and growth factor measurements from the mouse experiment described in Fig 1 with organoid protein expression data projected. Differentiated organoids project with focal inflammation and control groups, while proliferative, stem-like organoids are projected with the continuous inflammation group. Original samples used to generate the model project into the gray rectangle (control), blue rectangle (focal inflammation), and red rectangle (continuous inflammation). Underlying numerical values for panels A–C are provided in S1 Data. MCP-1, monocyte chemoattractant protein 1; MIG, mitokine induced by gamma interferon; MIP-1α, macrophage inflammatory protein 1-alpha.

Although our in vivo results were consistent with a model in which undifferentiated epithelium supplies many of the cytokines needed to recruit inflammatory cells to the colonic lamina propria, these experiments did not rule out the possibility that these cytokine and chemokine changes are secondary effects originating from immune cells. In order to test whether the expression of proinflammatory signaling molecules is intrinsic to the epithelium, we measured the production of cytokines and chemokines as a function of epithelial differentiation state in an in vitro 3D organoid model. Crypts were isolated from WT mouse colons and developed into 3D organoids in matrigel following well-established protocols [17]. We then shifted the growth conditions to maintain a mixed cell population to enrich for proliferative stem cells or to differentiate into the enterocyte or goblet cell lineages (S14 Fig) [15]. Following confirmation of appropriate differentiation based on expression of cell type markers (Fig 7A), purified organoids were analyzed for expression of the suite of cytokines, chemokines, and growth factors that were measured in intact colons (S15 and S16 Figs). Relative to the stem cell-enriched and mixed population organoids, the majority of cytokines and chemokines were suppressed in organoids enriched for secretory and/or absorptive cells. For example, MCP-1 was reduced during differentiation in a lineage-independent manner, while VEGF and mitokine induced by gamma interferon (MIG) were reduced only when cells were differentiated into the goblet lineage (Fig 7B). Interestingly, MIP-1α, which was strongly induced in animals with inflammation (S5 Fig), was increased in expression when organoids were differentiated into the goblet lineage. Altogether, these data suggest that differentiation alters the cytokine and chemokine expression pattern of the colonic epithelium independent of immune cells.

To put the in vitro analysis of cytokine/chemokine expression into the context of our prior in vivo studies, we projected the organoid expression data onto the same PLSR model from Fig 5D, where expression of the cytokines and chemokines highlighted in Fig 5C specifies latent variable 1 (LV1). In this analysis, the more stem-like organoids were projected positively on LV1, clustering with the inflamed mouse colons (Fig 7C). Conversely, the absorptive- and secretory-differentiated organoids clustered with the focal and noninflamed colons. This analysis demonstrates that the patterns of cytokine and chemokine expression that correlate with loss of differentiation and immune cell infiltration in inflamed colons in vivo are largely recapitulated in an in vitro system that lacks both bacteria and immune cells. Together with the in vivo mouse experiments, this result indicates that the colonic epithelium plays an active role in inflammation that extends beyond its role in maintaining barrier integrity.

## Discussion

The goals of this study were to define the tissue-level disease state in a mouse model of IBD and to use this information to understand how the components of the intestinal ecosystem interact to maintain homeostasis. We focused on the epithelium, the immune system, and the molecules that mediate interaction between them, using computational approaches to identify correlations between molecular, cellular, and histological characteristics of the inflammatory state in the TCT model. In doing so, we established a hypothesis-generating platform to identify pathways important for homeostasis and developed a composite phenotype that was used in subsequent experiments to measure the effects of pathway perturbation at a systems level (Fig 1).

Using our systems approach, we identified activation of the mTOR pathway as being one of the strongest correlates of colonic inflammation in this model (Fig 2). To determine whether this pathway plays an active role in the disease, we treated sick animals with rapamycin. Rapamycin (or sirolimus) is an inhibitor of the target of rapamycin complex 1 (TORC1) complex, but it should be noted that it can also exert mTOR-independent effects through its ability to bind FK506 binding protein 12 (FKBP12) [18]. Rapamycin treatment resulted in global changes in the inflammatory phenotype, including reduction of crypt hyperplasia, increased differentiation and decreased proliferation in the intestinal epithelium, decreased immune infiltration, and globally decreased expression of molecular correlates of inflammation (Fig 3). Notably, and contrary to prior observations [11, 19], rapamycin did not increase the number of Tregs in the colon of the TCT model (Fig 3D), nor did it affect the overall composition of the colonic microbiome (S11 Fig). While the role of mTOR signaling has not been studied in the TCT model of CD, several studies have demonstrated efficacy for rapamycin and related molecules (rapalogs) in mouse models of colitis, including chronically dextran sodium sulfate (DSS)-treated and IL-10 knockout mice [7, 20, 21], suggesting that mTOR might be a therapeutic target for IBD. In addition to our mouse studies, we also found that a subset of human IBD patients—those with ileal CD—had increased mTOR pathway activity in inflamed tissue (S9 Fig). Nevertheless, mTOR inhibitors have shown limited efficacy in unselected clinical trials for adult IBD patients [22], although rapamycin has shown clinical efficacy in individual adult cases and in a larger study of pediatric IBD patients [23, 24]. A clinical trial on patients selected for high mTOR signaling, perhaps using p-S6 as a biomarker, will be required to determine whether mTOR inhibition has potential as an effective therapeutic strategy for some IBD patients.

The identification of mTOR signaling as a mediator of colitis in the TCT model provided an entryway to study the cellular mechanisms controlling intestinal homeostasis. While mTOR has been linked to mouse colitis through the effect of rapamycin, the pathway was presumed to promote inflammation by affecting T helper cell proliferation and polarization, and the role of epithelial mTOR was not explored [7, 21]. By contrast, one key facet of the inflammatory phenotype that we found to be controlled by mTOR was loss of differentiation and expansion of the epithelial proliferative zone. Defective epithelial differentiation is a feature of IBD in human patients; goblet cell depletion has been reported in both CD and UC and has been linked to reduced cytokine secretion and antigen presentation [25, 26]. In our acute treatment experiments, we found that epithelial differentiation was rapidly induced by rapamycin and that this preceded changes in cytokines and chemokines, which preceded changes in innate immune infiltration (Fig 4). These data support a mechanism whereby induction of differentiation results in reduced chemokine signaling and reduced inflammatory infiltrate. The role that epithelial differentiation plays in protection from colitis has been explored in other mouse models. For example, mice in which differentiation of goblet cells is suppressed, such as those lacking Jak3 or overexpressing Claudin-1, are predisposed to spontaneous and DSS-induced colitis [27, 28]. Although goblet cells are important for establishing the barrier, these studies failed to recognize the potential for signaling interaction between the epithelial and immune components of the intestinal environment.

To test directly whether cellular differentiation state, rather than mTOR activation state, controlled the cytokine profile of the colon, we used the Notch inhibitor DBZ to drive goblet cell differentiation in inflamed epithelium, and this was also associated with decreased cytokine and chemokine expression and immune infiltrate (Fig 5). Pharmacologic inhibition of Notch is known to protect against DSS-induced colitis, and genetic inhibition of Notch was found to decrease the expression of proinflammatory cytokines in cultured Caco-2 cells [29, 30]. Notch signaling is the primary regulator of goblet cell differentiation, and most of the studies linking differentiation to colitis have focused on Notch. Interestingly, although Tsc2, an upstream regulator of mTOR signaling, has also been shown to regulate Notch [31], we could not find any evidence in our protein or gene expression data indicating that Notch signaling, in addition to mTOR signaling, is activated in the TCT model of colitis. This observation suggests that mTOR is affecting differentiation independently of Notch in the TCT model. Our transcriptional profiling analysis suggested that inflammation-associated mTOR activation is associated with an expansion of the transit amplifying pool (Fig 6), consistent with prior observations that mTOR signaling is important for repair of the colonic epithelium following damage, a process that is driven by TACs [32, 33]. Together, these observations are consistent with our model linking differentiation, and not mTOR or Notch signaling directly, to proinflammatory signaling by the epithelium.

Inhibition of both mTOR and Notch can affect the function of lymphocytes directly [34, 35], so our in vivo studies could not rule out a contribution of perturbation of immune cell function to the overall suppression of inflammation in animals treated with inhibitors. To address this limitation, we used an in vitro organoid system to investigate the relationship between differentiation state and cytokine secretion in epithelium that is isolated from the microbiome and the immune system. We found that proliferative organoids expressed higher levels of inflammation-associated cytokines, chemokines, and growth factors than either absorptive- or secretory-differentiated organoids (Fig 7). This result demonstrates that, even in the absence of gut bacteria and immune cells, the differentiation state of the epithelium, and not necessarily the activation state of mTOR or Notch, specifies the expression of immunomodulatory molecules.

Altogether, our work clarifies the important role that epithelial differentiation and epithelium-immune cross talk plays in maintaining overall colonic homeostasis. We demonstrate that undifferentiated epithelium plays an active role in maintaining inflammation by secreting chemokines that recruit innate immune cells such as macrophages and neutrophils. By extension, altered epithelial homeostasis is a central feature of self-perpetuating inflammation in the colon; since expansion of TACs is required for epithelial repair, the repair that serves to restore barrier function during acute inflammation will also function to recruit additional inflammatory cells to the site of damage. Notably, modulation of the proliferative state of the epithelium had minimal impact on T cell numbers within the tissue. In both mouse and human IBD, dysregulated T-cell activity is thought to be the key triggering event in inflammatory pathology. Our work suggests that T-cell activation is upstream of epithelial differentiation defects in the chain of events that leads to chronic inflammation. We propose that therapeutic approaches that promote epithelial differentiation could show efficacy in reducing chronic inflammation in IBD.

## Materials and methods

### TCT

TCT was performed according to established methods [8]. WT and Rag1-deficient mice on the C57BL/6 background were used for TCT. These animals were purchased from the Jackson Laboratory (Bar Harbor, Maine, United States). Naïve T cells and Tregs were collected on the BD Aria sorter and injected at 400,000 and 200,000 cells/animal, respectively.

### Ethics statement

All animal work was approved by the Institutional Care and Use Committees of Massachusetts General Hospital and Beth Israel Deaconess Medical Center under protocol numbers 2007N000058, 078–2014, and 080–2017. Approved protocols conformed to the USDA Animal Welfare Act, the PHS Policy on Humane Care and Use of Laboratory Animals, and the “ILAR Guide for the Care and Use of Laboratory Animals.”

### Inhibitor treatments

Rapamycin was purchased from LC Laboratories (R-5000), and treatment was carried out for 1, 4, 8, 24, or 48 hours for the acute experiment and for 2 weeks for the extended experiment. Rapamycin was injected IP at 5 mg/kg in a vehicle of 5.2% Tween 80 and 5.2% PEG400 in water. DBZ was purchased from Sigma (SML0649-25MG) and used at 10 μmol/kg in water with 0.5% HPMC and 0.1% Tween 80.

### Tissue processing and histology

Colons were removed and flushed with PBS, and one-fifth lengthwise was taken for flow cytometry. A matched fifth was cut into proximal and distal regions and lysed in 250 μl Bio-Plex lysis buffer with protease inhibitor, factors 1 and 2, and PMSF. The final piece was rolled and fixed overnight in 10% formalin for histology. For microarray tissue collection, one-fifth was snap frozen and processed as described below. This replaced flow cytometric measurement for 4 Treg and 6 inflamed animals from the initial study.

For crypt height measurements and gross histology, 5 μm sections of rolls were stained with hematoxylin and eosin (HE) according to standard protocols. Alcian Blue/Periodic Acid/Schiff was used from Leica (38016SS3A, 38016SS4A, and 38016SS4B), and goblet cells were visualized according to reagent instructions. Immunohistochemistry for phospho-Histone H3 S10 (Cell Signaling Technology 9701), liver fatty acid binding protein (Abcam ab7366), Ki-67 (Cell Signaling Technology 12202), chromogranin A (Abcam ab15160), and phospho-S6 ribosomal protein S235/236 (Cell Signaling Technology 4858) was carried out with citrate antigen retrieval and visualized using horseradish peroxidase reaction. For Foxp3 immunohistochemistry, antigen retrieval was performed in DAKO Target Retrieval Solution (#S1699) in a pressure cooker. Tissue sections were blocked with DAKO Serum-Free Protein Block (#X0909) and primary anti-E-cadherin (BD Medical Technology #BDB610181) and anti-FoxP3 (Abcam ab54501) antibodies were incubated overnight at 4°C in DAKO Antibody Diluent (#S3022). Following PBS washes, Alexa Fluor-labeled secondary antibodies (ThermoFisher) were incubated in DAKO Antibody Diluent for 1 hour at room temperature. Slides were mounted in ProLong mounting medium (ThermoFisher #P36962) and imaged on a Zeiss Axio Imager Z2.

Crypt height measurements were performed using an Olympus microscope or slide scanner software. Briefly, an arbitrary line was drawn from the crypt base to the top of the crypt every 5 crypts across the length of the colon. Crypt measurements in which multiple crypts were associated with a single line were deemed off axis and removed from subsequent analysis. For scatter plots, all measurements were evenly spaced across the distal and proximal regions separately using polyline measurements of the lengths of those sections. Averages were taken of all measurements in the distal and proximal regions separately. To calculate the proliferative zone, the distance from the crypt base to the highest PH3-positive cell or Ki-67-positive cell was divided by the total crypt height.

### Flow cytometry

Tissue was homogenized in serum-free DMEM with 2 mg/ml collagenase type I C (VWR 234153-100MG) and incubated for 1 hour at 37°C. Following incubation, the sample was strained through a 45-μm filter and spun down for 5 minutes at 700 g. Cells were then stained with the following antibodies from BioLegend (1:300 in fluorescence-activated cell sorting [FACS] buffer) for 10 minutes: Alexa-488 CD326 (118210), BV-421 F4/80 (123131), BV-605 CD4 (100547), BV-510 cd11b (101245), Alexa-700 Ly6G (127622), APC CD25 (102012), PE/Cy7 cd11c (117317), APC/Cy7 CD45 (103116), and PE CD45RB (103308). Cells were analyzed on a 5-laser LSR II (Becton Dickson). Details of markers used to quantify individual cells types are presented in S2 Fig.

### Protein measurements

Luminex-based protein measurements were carried out according to manufacturer’s instructions, as we have reported previously [34, 36, 37]. The following kits from Bio-Rad were used: Group I Cytokine Assay (M60-009RDPD), Group II 9-plex (MD0-00000EL), and Group III Th17 panel (171-FA001M). BioplexPro phosphoprotein measurements were carried out with the following groupings: 10-plex: Akt Ser473 (171-V50001M), c-Jun Ser63 (171-V50003M), CREB Ser133 (171-V50028M), Erk1/2 Thr202/Tyr204, Thr185/Tyr187 (171-V50006M), GSK-3 Ser21/Ser9 (171-V50007M), Jnk Thr183/Tyr185 (171-V50011M), Mek1 Ser217/Ser221 (171-V50012M), p38 MAPK Thr180/Tyr182 (171-V50014M), Stat3 Ser727 (171-V50021M), p90Rsk Ser380 (171-V50035M); single-plex: IR-1 Tyr1146 (171-V50031M), IRS-1 Ser636/Ser639 (171-V50030M), IκB Ser32/Ser36 (171-V50010M), p70S6K Thr389 (171-V50016M), S6RP Ser235/Ser236 (171-V50038M), and Atf-2 Thr71 (171-V50024M).

### Colonic organoid culture

Crypt epithelial cells were isolated from mouse colons by incubating the tissue in 2 mg/ml type I collagenase (Invitrogen). Organoids were grown in matrigel in the presence of EGF, Noggin, R-spondin, and Wnt3a as previously described [17, 38]. During the first 4 days of culture, GSK3β inhibitor, CHIR99021 (3 μM, Stemgent), and histone deacetylase inhibitor and Notch agonist, VPA (1 mM, Sigma-Aldrich), were added to the medium to enrich for stem cells. Following this treatment, organoids were either maintained in this stem cell media, returned to standard growth media, or differentiated as previously described with the following minor modifications [17]. To increase enterocyte differentiation, organoids were grown in Wnt3a-deficient medium supplemented with VPA (1 mM) and the Wnt inhibitor IWP-2 (5μm, Stemgent). To promote goblet cell differentiation, Wnt3a-deficient medium was supplemented with the Notch inhibitor DAPT (10 μm, Stemgent) and IWP2. Following 4 additional days of treatment, organoids were harvested for RNA collection or lysed for Luminex analysis.

### Microarray analysis and GSEA

RNA was harvested from snap-frozen colon tissue using the Qiagen RNAeasy microarray tissue mini kit (cat. 73304). Microarray was performed using Affymetrix mouse 430 2.0 GeneChip at the Dana Farber microarray core. Microarray data were deposited in the NCBI gene expression omnibus (accession number GSE87317).

The following gene sets were pulled and generated from published manuscripts: Atoh1 null, secretory progenitors (Sec-pro), and enterocytes [15]; Lgr5-positive cells [14]; and TACs [16]. All gene sets were used as published except the enterocyte gene set, which was generated using the raw data from Kim et al. Enterocyte-specific genes were defined as the transcripts that showed at least 2-fold increased expression compared to all other measured cell types (Lgr5, Atoh1 null, and Sec-pro) with an FDR < 0.05. Gene set enrichment analysis was run using the Broad Institute’s GSEA software [39].

### Microbiome analysis

Mice were treated with rapamycin or vehicle daily for 2 weeks as described above. Fecal samples were harvested pretreatment and post-treatment, frozen until the end of the treatment period, and then homogenized in DNA/RNA Shield reagent (Zymo R1100-50) at 10% (v/v). DNA was extracted using the ZymoBIOMICS DNA Miniprep Kit (Zymo R2002). Generation of the 16s library, sequencing, read calling, and taxonomy assignment were performed by Zymo Research through their ZymoBIOMICS service. ZymoBIOMICS Microbial Community Standards (Zymo D6300) were used as positive controls. The library was sequenced on an Illumina MiSeq with >10% PhiX spike-in. Amplicon sequences were deduced from raw reads, and chimeric sequences were removed using the Dada2 pipeline [40]. Taxonomy assignment was performed using Qiime v1.9.1 [41]. PCoA and correlation analysis of Bray-Curtis dissimilarity scores were performed using MATLAB.

## Supporting information

S1 Data Raw numerical values for data presented in manuscript. (XLSX)

S1 Fig Experimental setup and tissue processing work flow. (A) Representative fluorescence-activated cell sorting (FACS) plots for isolation of CD45RBhi naive T cells and CD25+ regulatory T cells (Tregs). (B) Time course of induction and assessment of inflammation. Rag1 null animals are injected with naïve T cells or Tregs and assessed for signs of inflammation such as weight loss. When the first animals began to show sustained weight loss, the remaining animals were killed at various time points and processed as described in panel C. (C) Tissue processing strategy. Colons were resected and opened longitudinally. Approximately one-sixth was taken for flow cytometry. Matched tissue was lysed for Luminex analysis after splitting into proximal and distal regions. The remaining tissue was rolled (see hematoxylin–eosin [HE] at right side of panel), allowing for visualization of the entire colon in a single section.
(PDF)

S2 Fig Phenotypic characterization of T-cell transfer (TCT) mice. (A) Cellular phenotypes during colitis. Mitotic cells were detected by immunohistochemistry for phosphorylated histone H3 (PH3). While restricted to the lower crypts in normal colons, mitotic cells extend to the luminal surface during colitis. Alcian Blue/Periodic Acid Schiff (AB/PAS) staining was used to stain mucins in the goblet cell cytoplasm. Secretory enteroendocrine cells and absorptive enterocytes were detected by staining for chromogranin A (Chga) and liver fatty acid binding protein (Fabpl). Both cell types are largely absent in animals with colitis. (B) Quantification of the number of epithelial cells per crypt. In this experiment, the total number of cells per crypt in a single cross section was counted, only for crypts in which the entire crypt was sectioned. *N* = 120 crypts for both regulatory T cell (Treg) control and for the experimental naive T recipients. The *p*-value was determined by Mann-Whitney test. (C) Immunohistochemistry (IHC) for mast cell tryptase. The number of mast cells in the control and inflamed colons was too low to quantify. Spleen from wild-type animals was used as a positive control. Underlying numerical values for panel B are provided in S1 Data.
(PDF)

S3 Fig Immune phenotypes associated with inflammation. (A) Representative fluorescence-activated cell sorting (FACS) plots showing strategies for quantifying different immune cell populations. (B) FACS-based quantification of the composition of colonic immune infiltrate for animals of the 3 phenotypic classes. Horizontal and vertical lines represent mean +/− standard error. Underlying numerical values for panel B are provided in S1 Data.
(PDF)

S4 Fig Phosphoproteins measured by Luminex. Proteins were measured from the distal colons of control and naïve T-treated animals and those treated for 2 weeks with vehicle or rapamycin. Data are represented in arbitrary relative fluorescence units (RFUs). Significance was determined by *t* test (squares) or one-way ANOVA with Tukey post-test (circles). *P*-values are denoted by * *p* < 0.05, ** *p* < 0.005, and *** *p* < 0.001. Underlying numerical values are provided in S1 Data.
(PDF)

S5 Fig Chemokines and growth factors measured by Luminex. Proteins were measured from the distal colons of control and naïve T-treated animals and those treated for 2 weeks with vehicle or rapamycin. Data are presented as absolute concentration from the tissue (pg/ml). Significance determined by *t* test (squares) or one-way ANOVA with Tukey post-test (circles). *P*-values are denoted by * *p* < 0.05, ** *p* < 0.005, and *** *p* < 0.001. Underlying numerical values are provided in S1 Data.
(PDF)

S6 Fig Interleukins and cytokines measured by Luminex. Proteins were measured from the distal colons of control and naïve T-treated animals and those treated for 2 weeks with vehicle or rapamycin. Data are presented as absolute concentration from the tissue (pg/ml). Significance was determined by *t* test (squares) or one-way ANOVA with Tukey post-test (circles). *P*-values are denoted by * *p* < 0.05, ** *p* < 0.005, and *** *p* < 0.001. Underlying numerical values are provided in S1 Data.
(PDF)

S7 Fig Interleukins and cytokines measured by Luminex. Proteins were measured from the distal colons of wild-type C57BL/6J, Rag1 null (control), regulatory T cell (Treg)-injected, and naïve T-injected animals. In this experiment, animals injected with Tregs or naïve T cells were aged for only 4 weeks after adoptive transfer. This is a time point at which animals receiving naïve T cells do not yet have inflammation. Data are presented as absolute concentration from the tissue (pg/ml). None of the intergroup comparisons reached statistical significance. Underlying numerical values are provided in S1 Data.
(PDF)

S8 Fig Induction of mammalian target of rapamycin (mTOR) pathway activity, as measured by p-S6 ribosomal protein (p-S6RP), in multiple mouse models of inflammatory bowel disease (IBD). The genotype of each group is indicated below the measurements: +/− for heterozygous and −/− for null. Dhet, double heterozygous; DKO, double knockout. The genetic background of each model is indicated in parentheses. p-S6RP was measured via Luminex assay. Horizontal and vertical lines represent mean +/− standard error. Significance was determined by one-way ANOVA with Tukey-Kramer post-test. Underlying numerical values are provided in S1 Data.
(PDF)

S9 Fig Mammalian target of rapamycin (mTOR) activation in human inflammatory bowel disease (IBD) patients. Matched biopsies from active inflammation and noninvolved regions were taken from individuals with Crohn’s disease and ulcerative colitis. mTOR activity was assessed by measuring phosphorylation of its downstream target S6 ribosomal protein and is depicted as fold change of involved versus noninvolved on a patient-by-patient basis. Overall, there was not a clear trend towards increased mTOR activity in inflamed regions; however, the subset of patients with ileal Crohn’s disease showed consistent up-regulation of mTOR signaling in regions of inflammation. The *p*-value was determined by unpaired *t* test. Underlying numerical values are provided in S1 Data.
(PDF)

S10 Fig Expression of cytokines, chemokines, and growth factors following acute rapamycin treatment. Individual measurements for inflammation-associated cytokines were determined by Luminex analysis. Plots represent mean and standard error. Underlying numerical values are provided in S1 Data.
(PDF)

S11 Fig Microbiome analysis of animals with inflammation. (A) Microbial composition plots showing distribution of phyla and genera from mice before and after treatment with rapamycin. Each pair of columns represents the composition of fecal pellets taken from the same mouse before and after treatment. (B) Heat map of Bray-Curtis dissimilarity scores from all samples. (C) Principle coordinate analysis (PCoA) plot created using the matrix of paired-wise distance between samples calculated by the Bray-Curtis dissimilarity. Each dot on the figure represents the whole microbial composition profile. Samples are color-coded based on treatment group. Underlying numerical values are provided in S1 Data.
(PDF)

S12 Fig Expression of cytokines, chemokines, and growth factors following dibenzazepine (DBZ) treatment. Individual measurements for each cytokine, chemokine, and growth factor represented in Fig 5C. Plots represent mean +/− standard error. Significance was determined by ANOVA with Tukey-Kramer post-test. * represents *p* ≤ 0.05; ** represents *p* ≤ 0.01; *** represents *p* ≤ 0.001. Underlying numerical values are provided in S1 Data.
(PDF)

S13 Fig Expression of cytokines, chemokines, and growth factors following dibenzazepine (DBZ) treatment. Individual measurements for each cytokine, chemokine, and growth factor represented in Fig 5C. Plots represent mean +/− standard error. Significance was determined by ANOVA with Tukey-Kramer post-test. * represents *p* ≤ 0.05; ** represents *p* ≤ 0.01; *** represents *p* ≤ 0.001. Underlying numerical values are provided in S1 Data.
(PDF)

S14 Fig Organoid culture conditions for enriching various cell populations. Treating established organoids with various drug combinations enriches for stem cells, enteroctyes, or goblet cells. Wnt-conditioned media contain EGF, Noggin, and R-spondin as well supernatants from a Wnt3A overexpressing cell line. CHIR99021 is a GSK3 inhibitor, and valproic acid (VPA) is a histone deacetylase inhibitor and Notch pathway activator. IWP-2 is a Wnt inhibitor, and DAPT is a γ-secretase inhibitor that blocks Notch pathway activity (see Materials and methods).
(PDF)

S15 Fig Expression of cytokines, chemokines, and growth factors in organoids. Individual measurements for each cytokine, chemokine, and growth factor represented in Fig 7B. Analytes with maximum expression values of <0.1 pg/ml were omitted. Plots represent mean +/− standard error. Significance was determined by ANOVA with Tukey-Kramer post-test. * represents *p* ≤ 0.05; ** represents *p* ≤ 0.01; *** represents *p* ≤ 0.001. Underlying numerical values are provided in S1 Data.
(PDF)

S16 Fig Expression of cytokines, chemokines, and growth factors in organoids. Individual measurements for each cytokine, chemokine, and growth factor represented in Fig 7B. Analytes with a maximum expression of <0.1 pg/ml were omitted. Plots represent mean +/− standard error. Significance was determined by ANOVA with Tukey-Kramer post-test. * represents *p* ≤ 0.05; ** represents *p* ≤ 0.01; *** represents *p* ≤ 0.001. Underlying numerical values are provided in S1 Data.
(PDF)

## Abbreviations

CD: Crohn’s disease
DBZ: dibenzazepine
DSS: dextran sodium sulfate
FACS: fluorescence-activated cell sorting
FKBP12: FK506 binding protein 12
GSEA: Gene Set Enrichment Analysis
GWAS: genome-wide association study
HE: hematoxylin–eosin
IBD: inflammatory bowel disease
IL: interleukin
IP: intraperitoneal
IRS1: insulin receptor substrate 1
KC: keratinocyte chemoattractant
LIF: leukemia inhibitory factor
LV1: latent variable 1
MCP-1: monocyte chemoattractant protein 1
MIG: mitokine induced by gamma interferon
MIP: macrophage inflammatory protein
mTOR: mammalian target of rapamycin
PC1: principal component 1
PCA: principal component analysis
PCoA: principal coordinate analysis
pDC: plasmacytoid dendritic cell
PLSR: partial least squares regression
p-S6: phosphorylated S6
qPCR: quantitative polymerase chain reaction
RANTES: regulated on activation, normal T cell expressed and secreted
SNP: single nucleotide polymorphism
TAC: transit-amplifying cell
TCT: T-cell transfer
Th2: T helper cell 2
TNF-α: tumor necrosis factor alpha
TORC1: target of rapamycin complex 1
Treg: regulatory T cell
UC: ulcerative colitis
VEGF: vascular endothelial growth factor
WT: wild-type
