# Supplementary material for: The colonic epithelium plays an active role in promoting colitis by shaping the tissue cytokine profile
Source: PLoS Biol. 2018 Mar 29;16(3):e2002417. doi: 10.1371/journal.pbio.2002417 (PMC5892915; doi:10.1371/journal.pbio.2002417)
Supplement: S1 Fig — (A) Representative fluorescence-activated cell sorting (FACS) plots for isolation of CD45RBhi naive T cells and CD25+ regulatory T cells (Tregs). (B) Time course of induction and assessment of inflammation. Rag1 null animals are injected with naïve T cells or Tregs and assessed for signs of inflammation such as weight loss. When the first animals began to show sustained weight loss, the remaining animals were killed at various time points and processed as described in panel C. (C) Tissue processing strategy. Colons were resected and opened longitudinally. Approximately one-sixth was taken for flow cytometry. Matched tissue was lysed for Luminex analysis after splitting into proximal and distal regions. The remaining tissue was rolled (see hematoxylin–eosin [HE] at right side of panel), allowing for visualization of the entire colon in a single section. (PDF) [file pbio.2002417.s002.pdf]

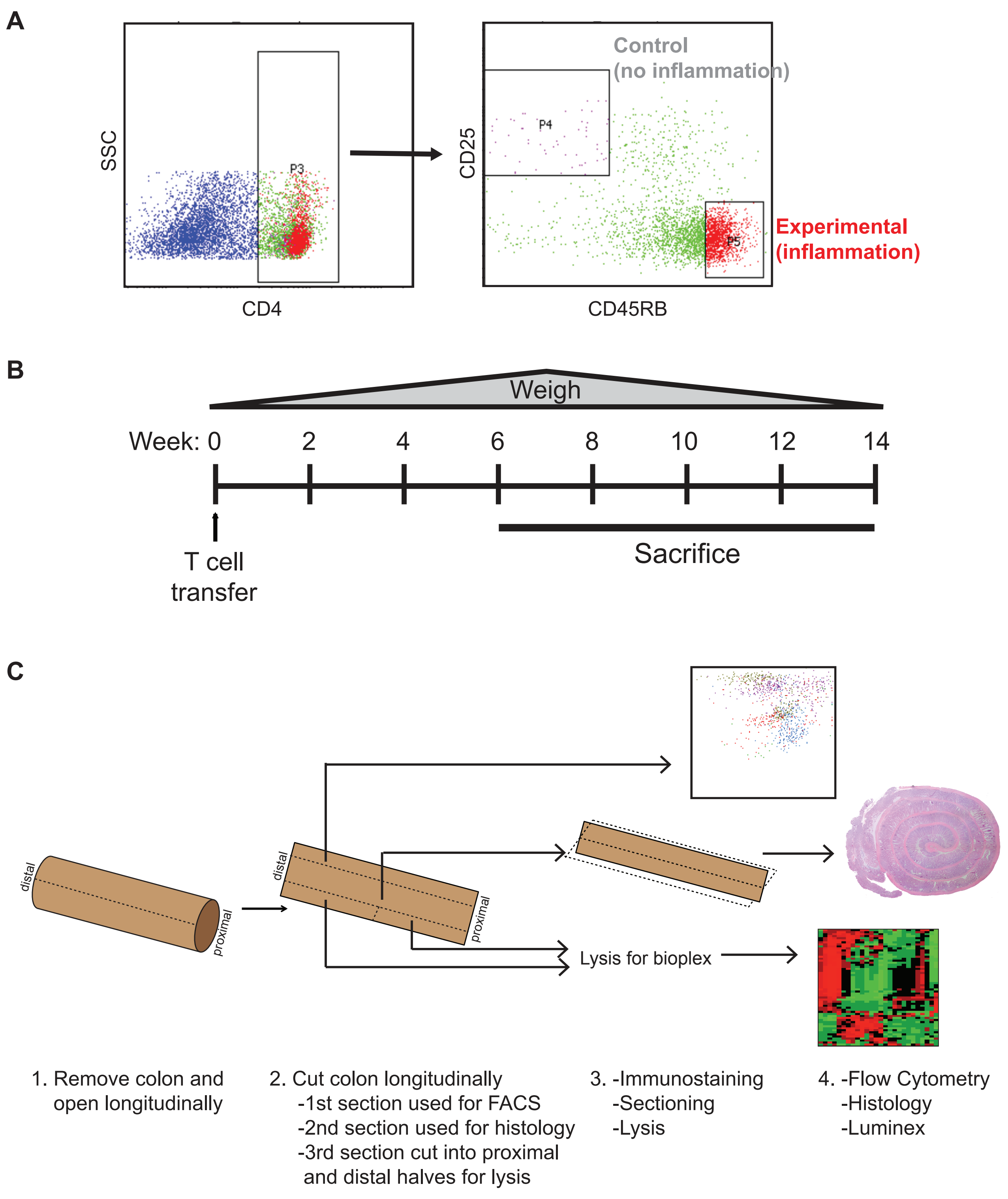

S1 Fig. Experimental setup and tissue processing work flow. (A) Representative fluorescence-activated cell sorting (FACS) plots for isolation of CD45RBhi naive T cells and CD25+ regulatory T cells (Tregs). (B) Time course of induction and assessment of inflammation. Rag1 null animals are injected with naïve T cells or Tregs and assessed for signs of inflammation such as weight loss. When the first animals began to show sustained weight loss, the remaining animals were killed at various time points and processed as described in panel C. (C) Tissue processing strategy. Colons were resected and opened longitudinally. Approximately one-sixth was taken for flow cytometry. Matched tissue was lysed for Luminex analysis after splitting into proximal and distal regions. The remaining tissue was rolled (see hematoxylin–eosin [HE] at right side of panel), allowing for visualization of the entire colon in a single section.
