# Supplementary material for: The colonic epithelium plays an active role in promoting colitis by shaping the tissue cytokine profile
Source: PLoS Biol. 2018 Mar 29;16(3):e2002417. doi: 10.1371/journal.pbio.2002417 (PMC5892915; doi:10.1371/journal.pbio.2002417)
Supplement: S2 Fig — (A) Cellular phenotypes during colitis. Mitotic cells were detected by immunohistochemistry for phosphorylated histone H3 (PH3). While restricted to the lower crypts in normal colons, mitotic cells extend to the luminal surface during colitis. Alcian Blue/Periodic Acid Schiff (AB/PAS) staining was used to stain mucins in the goblet cell cytoplasm. Secretory enteroendocrine cells and absorptive enterocytes were detected by staining for chromogranin A (Chga) and liver fatty acid binding protein (Fabpl). Both cell types are largely absent in animals with colitis. (B) Quantification of the number of epithelial cells per crypt. In this experiment, the total number of cells per crypt in a single cross section was counted, only for crypts in which the entire crypt was sectioned. N = 120 crypts for both regulatory T cell (Treg) control and for the experimental naive T recipients. The p-value was determined by Mann-Whitney test. (C) Immunohistochemistry (IHC) for mast cell tryptase. The number of mast cells in the control and inflamed colons was too low to quantify. Spleen from wild-type animals was used as a positive control. Underlying numerical values for panel B are provided in S1 Data. (PDF) [file pbio.2002417.s003.pdf]

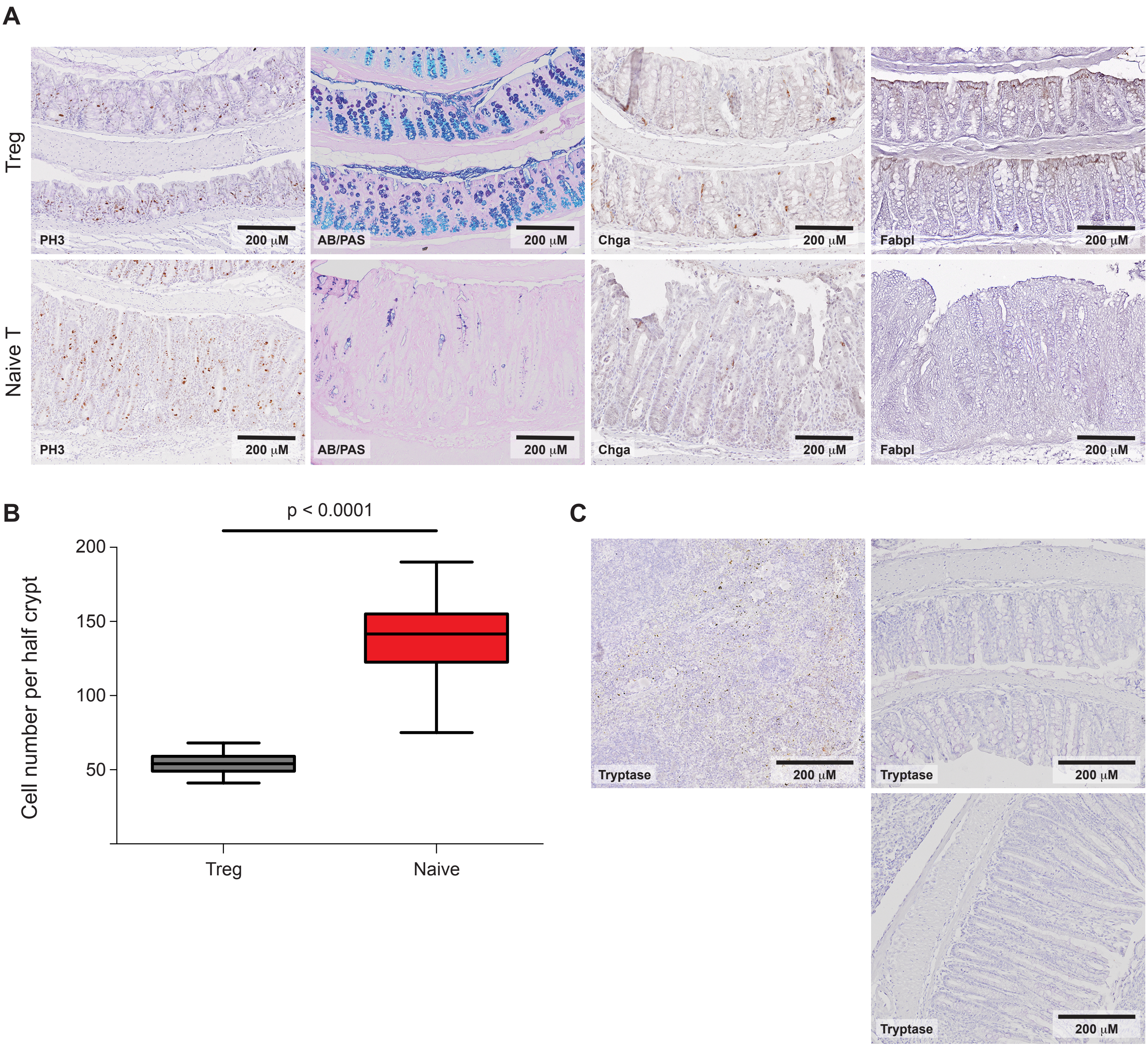

S2 Fig. Phenotypic characterization of T-cell transfer (TCT) mice. (A) Cellular phenotypes during colitis. Mitotic cells were detected by immunohistochemistry for phosphorylated histone H3 (PH3). While restricted to the lower crypts in normal colons, mitotic cells extend to the luminal surface during colitis. Alcian Blue/Periodic Acid Schiff (AB/PAS) staining was used to stain mucins in the goblet cell cytoplasm. Secretory enteroendocrine cells and absorptive enterocytes were detected by staining for chromogranin A (Chga) and liver fatty acid binding protein (Fabpl). Both cell types are largely absent in animals with colitis. (B) Quantification of the number of epithelial cells per crypt. In this experiment, the total number of cells per crypt in a single cross section was counted, only for crypts in which the entire crypt was sectioned. N = 120 crypts for both regulatory T cell (Treg) control and for the experimental naive T recipients. The p-value was determined by Mann-Whitney test. (C) Immunohistochemistry (IHC) for mast cell tryptase. The number of mast cells in the control and inflamed colons was too low to quantify. Spleen from wild-type animals was used as a positive control.

Underlying numerical values for panel B are provided in S1 Data.
