# Supplementary material for: The colonic epithelium plays an active role in promoting colitis by shaping the tissue cytokine profile
Source: PLoS Biol. 2018 Mar 29;16(3):e2002417. doi: 10.1371/journal.pbio.2002417 (PMC5892915; doi:10.1371/journal.pbio.2002417)
Supplement: S3 Fig — (A) Representative fluorescence-activated cell sorting (FACS) plots showing strategies for quantifying different immune cell populations. (B) FACS-based quantification of the composition of colonic immune infiltrate for animals of the 3 phenotypic classes. Horizontal and vertical lines represent mean +/− standard error. Underlying numerical values for panel B are provided in S1 Data. (PDF) [file pbio.2002417.s004.pdf]

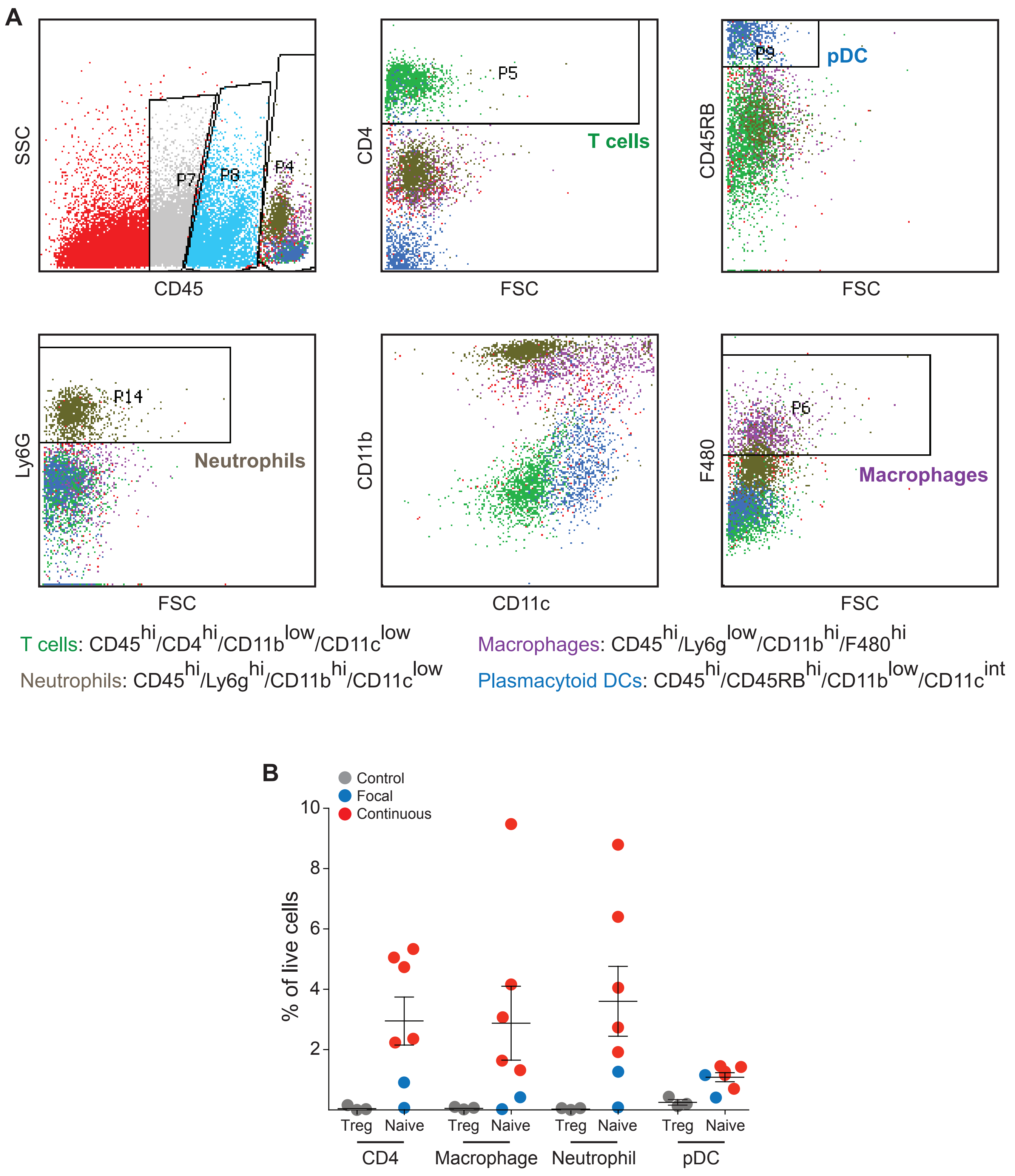

S3 Fig. Immune phenotypes associated with inflammation. (A) Representative fluorescence-activated cell sorting (FACS) plots showing strategies for quantifying different immune cell populations. (B) FACS-based quantification of the composition of colonic immune infiltrate for animals of the 3 phenotypic classes. Horizontal and vertical lines represent mean +/− standard error.
Underlying numerical values for panel B are provided in S1 Data.
