# Supplementary material for: The colonic epithelium plays an active role in promoting colitis by shaping the tissue cytokine profile
Source: PLoS Biol. 2018 Mar 29;16(3):e2002417. doi: 10.1371/journal.pbio.2002417 (PMC5892915; doi:10.1371/journal.pbio.2002417)
Supplement: S5 Fig — Proteins were measured from the distal colons of control and naïve T-treated animals and those treated for 2 weeks with vehicle or rapamycin. Data are presented as absolute concentration from the tissue (pg/ml). Significance determined by t test (squares) or one-way ANOVA with Tukey post-test (circles). P-values are denoted by * p < 0.05, ** p < 0.005, and *** p < 0.001. Underlying numerical values are provided in S1 Data. (PDF) [file pbio.2002417.s006.pdf]

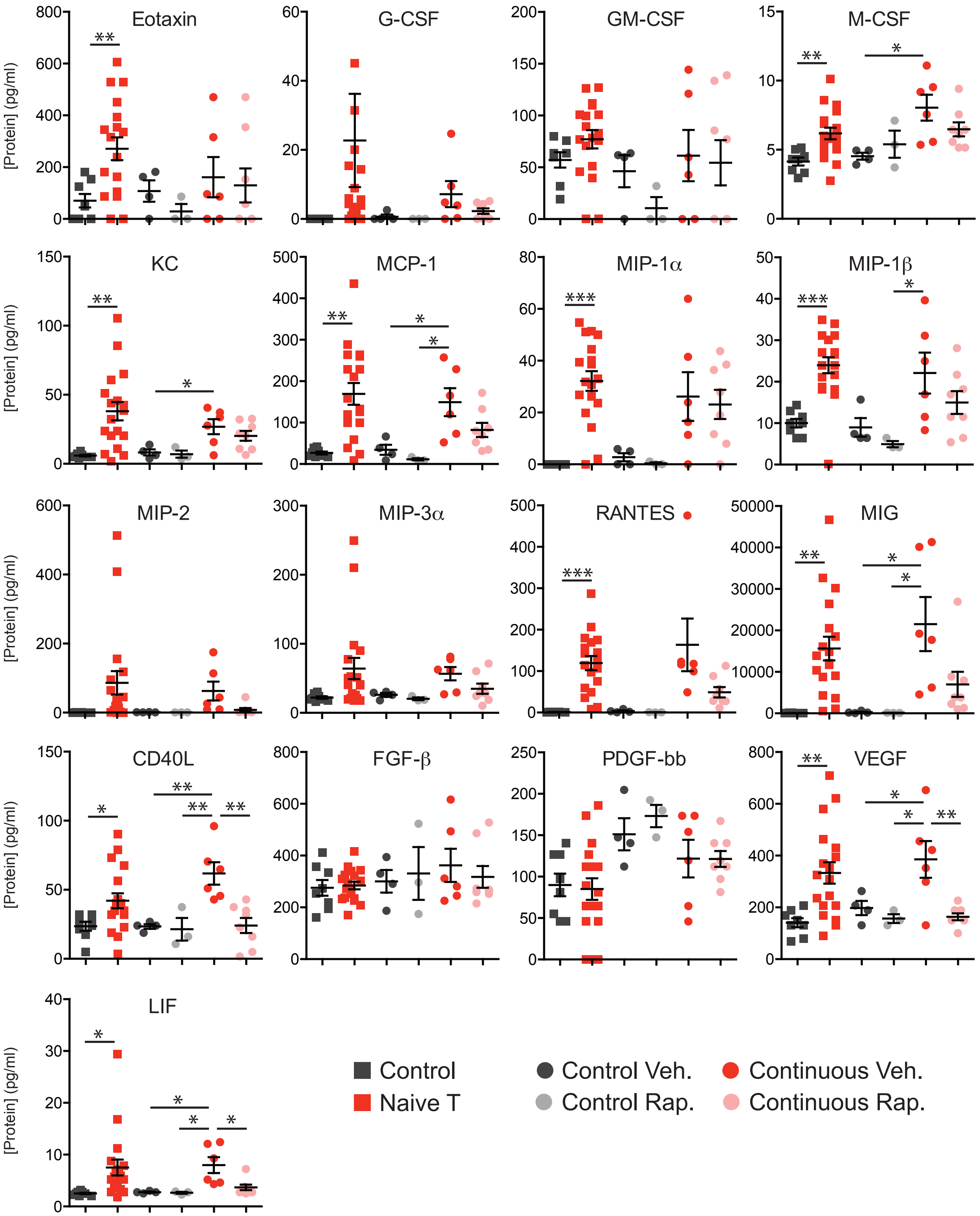

S5 Fig. Chemokines and growth factors measured by Luminex. Proteins were measured from the distal colons of control and naïve T-treated animals and those treated for 2 weeks with vehicle or rapamycin. Data are presented as absolute concentration from the tissue (pg/ml). Significance determined by t test (squares) or one-way ANOVA with Tukey post-test (circles). P-values are denoted by * $p < 0.05$, ** $p < 0.005$, and *** $p < 0.001$.

Underlying numerical values are provided in S1 Data.
