# Supplementary material for: The colonic epithelium plays an active role in promoting colitis by shaping the tissue cytokine profile
Source: PLoS Biol. 2018 Mar 29;16(3):e2002417. doi: 10.1371/journal.pbio.2002417 (PMC5892915; doi:10.1371/journal.pbio.2002417)
Supplement: S7 Fig — Proteins were measured from the distal colons of wild-type C57BL/6J, Rag1 null (control), regulatory T cell (Treg)-injected, and naïve T-injected animals. In this experiment, animals injected with Tregs or naïve T cells were aged for only 4 weeks after adoptive transfer. This is a time point at which animals receiving naïve T cells do not yet have inflammation. Data are presented as absolute concentration from the tissue (pg/ml). None of the intergroup comparisons reached statistical significance. Underlying numerical values are provided in S1 Data. (PDF) [file pbio.2002417.s008.pdf]

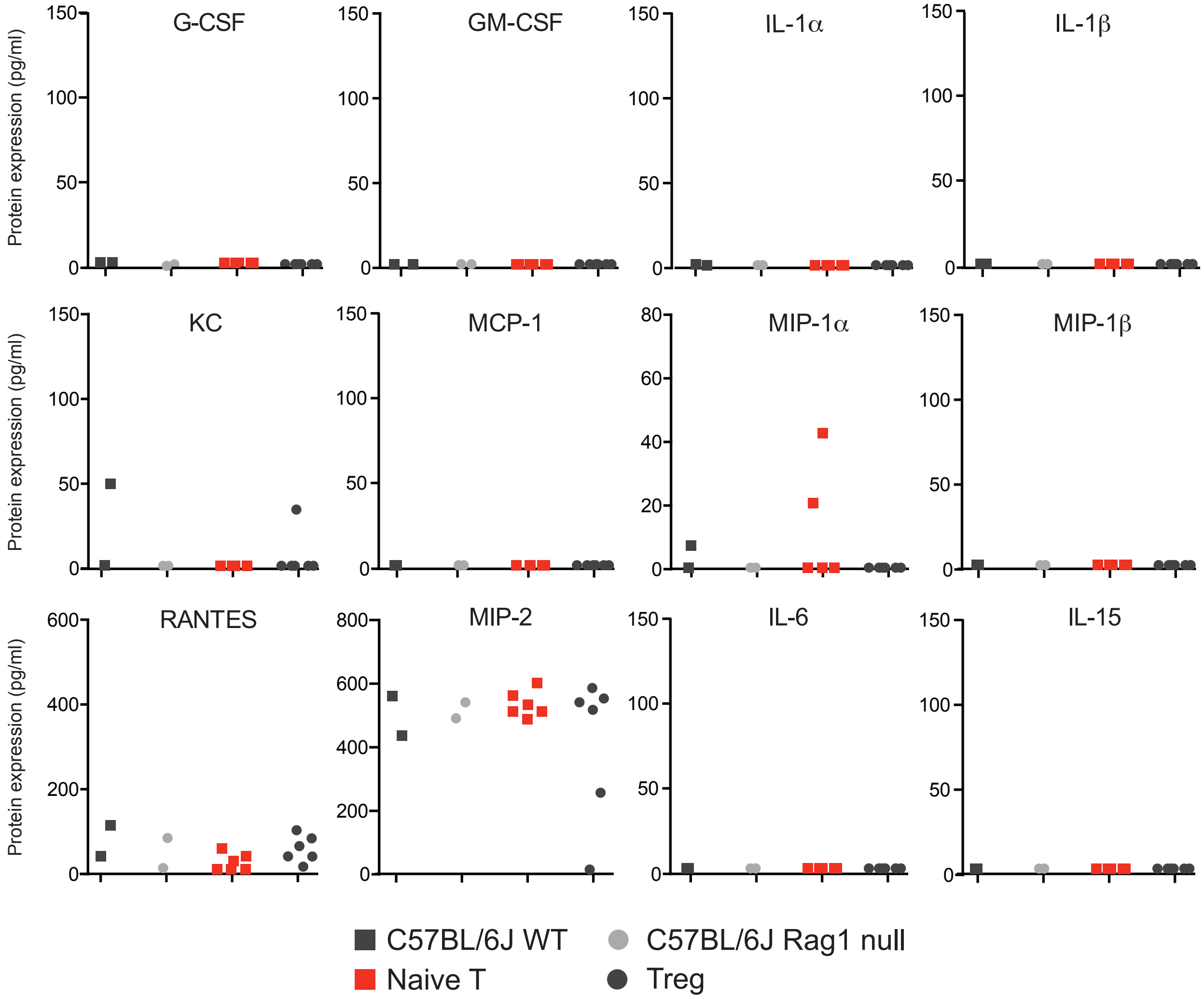

S7 Fig. Interleukins and cytokines measured by Luminex. Proteins were measured from the distal colons of wild-type C57BL/6J, Rag1 null (control), regulatory T cell (Treg)-injected, and naïve T-injected animals. In this experiment, animals injected with Tregs or naïve T cells were aged for only 4 weeks after adoptive transfer. This is a time point at which animals receiving naïve T cells do not yet have inflammation. Data are presented as absolute concentration from the tissue (pg/ml). None of the intergroup comparisons reached statistical significance.

Underlying numerical values are provided in S1 Data.
