# Supplementary material for: The colonic epithelium plays an active role in promoting colitis by shaping the tissue cytokine profile
Source: PLoS Biol. 2018 Mar 29;16(3):e2002417. doi: 10.1371/journal.pbio.2002417 (PMC5892915; doi:10.1371/journal.pbio.2002417)
Supplement: S8 Fig — The genotype of each group is indicated below the measurements: +/− for heterozygous and −/− for null. Dhet, double heterozygous; DKO, double knockout. The genetic background of each model is indicated in parentheses. p-S6RP was measured via Luminex assay. Horizontal and vertical lines represent mean +/− standard error. Significance was determined by one-way ANOVA with Tukey-Kramer post-test. Underlying numerical values are provided in S1 Data. (PDF) [file pbio.2002417.s009.pdf]

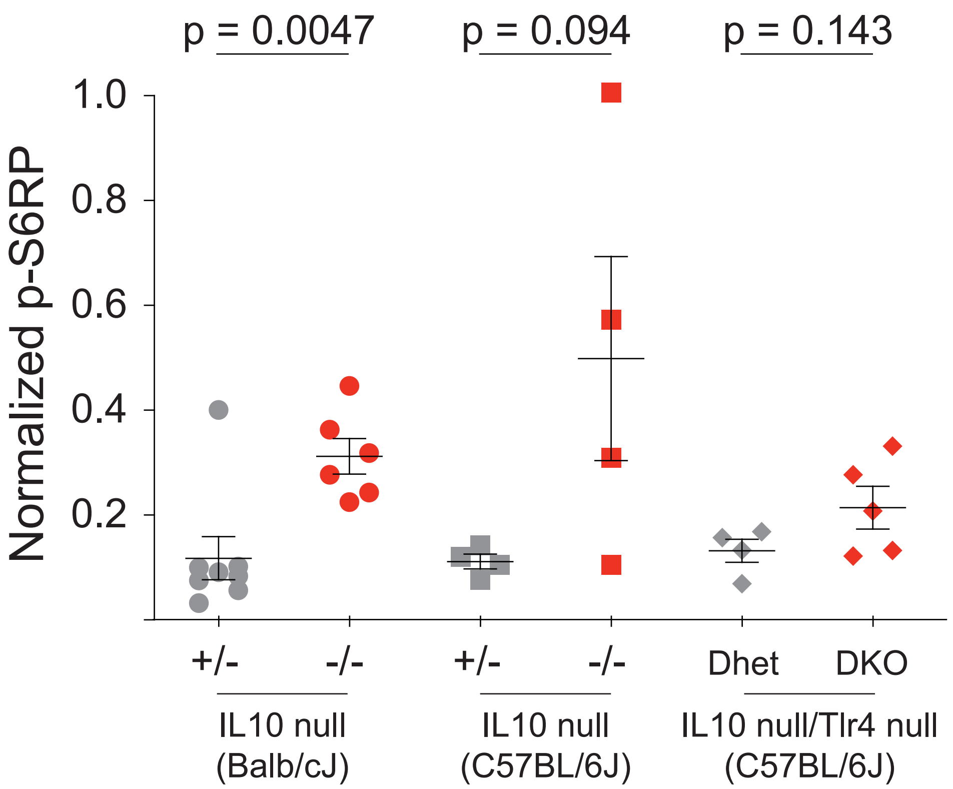


**S8 Fig. Induction of mammalian target of rapamycin (mTOR) pathway activity, as measured by p-S6 ribosomal protein (p-S6RP), in multiple mouse models of inflammatory bowel disease (IBD).** The genotype of each group is indicated below the measurements: +/− for heterozygous and −/− for null. Dhet, double heterozygous; DKO, double knockout. The genetic background of each model is indicated in parentheses. p-S6RP was measured via Luminex assay. Horizontal and vertical lines represent mean +/− standard error. Significance was determined by one-way ANOVA with Tukey-Kramer post-test.

Underlying numerical values are provided in S1 Data.
