# Supplementary material for: The colonic epithelium plays an active role in promoting colitis by shaping the tissue cytokine profile
Source: PLoS Biol. 2018 Mar 29;16(3):e2002417. doi: 10.1371/journal.pbio.2002417 (PMC5892915; doi:10.1371/journal.pbio.2002417)
Supplement: S9 Fig — Matched biopsies from active inflammation and noninvolved regions were taken from individuals with Crohn’s disease and ulcerative colitis. mTOR activity was assessed by measuring phosphorylation of its downstream target S6 ribosomal protein and is depicted as fold change of involved versus noninvolved on a patient-by-patient basis. Overall, there was not a clear trend towards increased mTOR activity in inflamed regions; however, the subset of patients with ileal Crohn’s disease showed consistent up-regulation of mTOR signaling in regions of inflammation. The p-value was determined by unpaired t test. Underlying numerical values are provided in S1 Data. (PDF) [file pbio.2002417.s010.pdf]

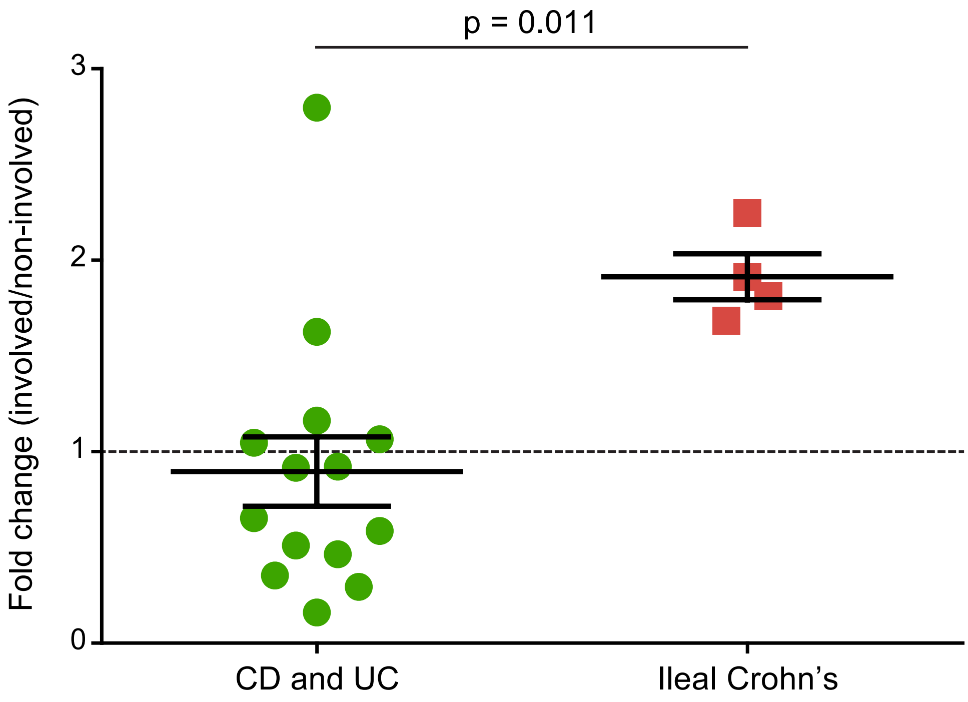

S9 Fig. Mammalian target of rapamycin (mTOR) activation in human inflammatory bowel disease (IBD) patients. Matched biopsies from active inflammation and noninvolved regions were taken from individuals with Crohn disease and ulcerative colitis. mTOR activity was assessed by measuring phosphorylation of its downstream target S6 ribosomal protein and is depicted as fold change of involved versus noninvolved on a patient-by-patient basis. Overall, there was not a clear trend towards increased mTOR activity in inflamed regions; however, the subset of patients with ileal Crohn disease showed consistent up-regulation of mTOR signaling in regions of inflammation. The p-value was determined by unpaired t test. Underlying numerical values are provided in S1 Data.
