# Supplementary material for: The colonic epithelium plays an active role in promoting colitis by shaping the tissue cytokine profile
Source: PLoS Biol. 2018 Mar 29;16(3):e2002417. doi: 10.1371/journal.pbio.2002417 (PMC5892915; doi:10.1371/journal.pbio.2002417)
Supplement: S10 Fig — Individual measurements for inflammation-associated cytokines were determined by Luminex analysis. Plots represent mean and standard error. Underlying numerical values are provided in S1 Data. (PDF) [file pbio.2002417.s011.pdf]

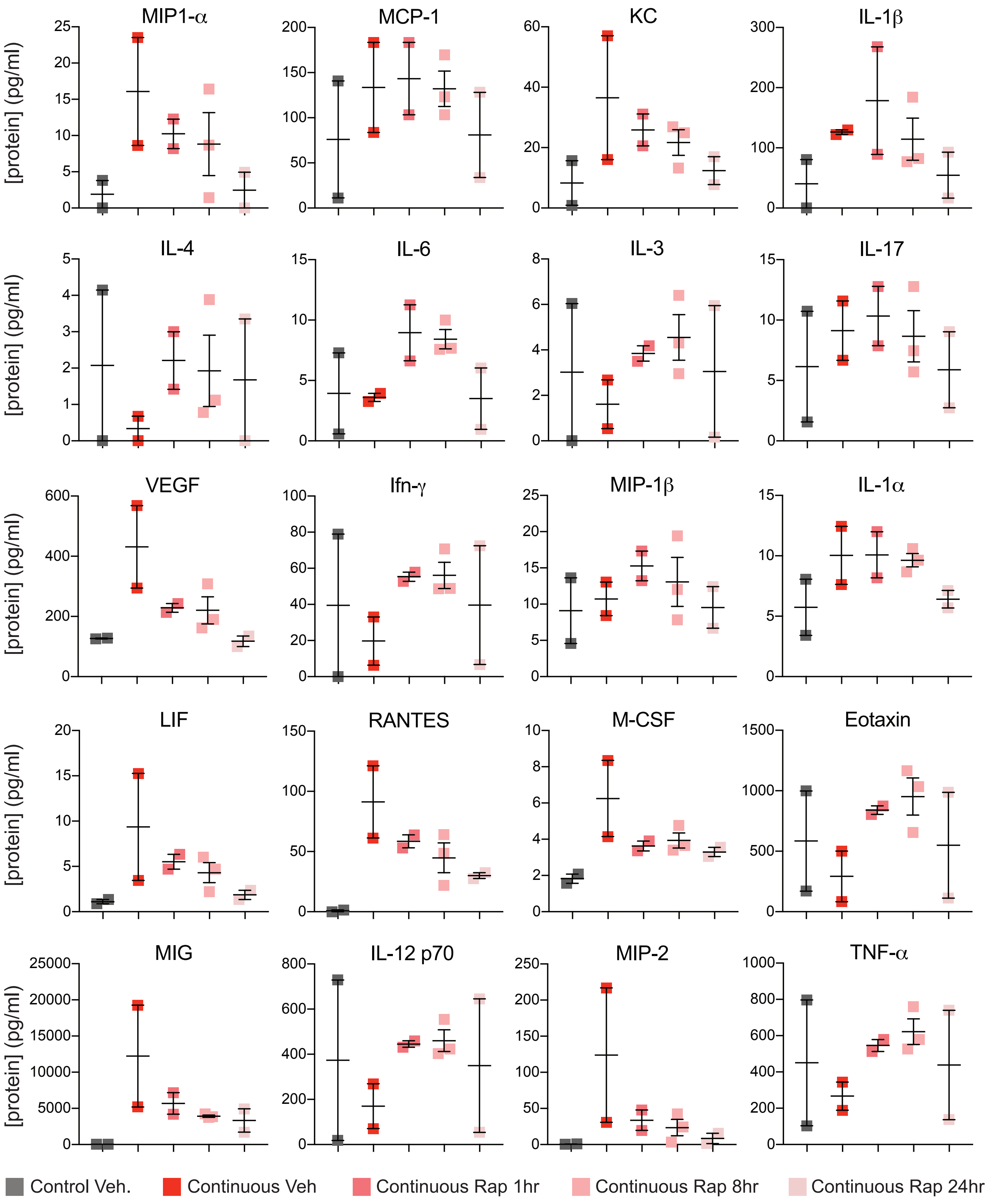


**S10 Fig. Expression of cytokines, chemokines, and growth factors following acute rapamycin treatment.** Individual measurements for inflammation-associated cytokines were determined by Luminex analysis. Plots represent mean and standard error.

Underlying numerical values are provided in S1 Data.
