# Supplementary material for: The colonic epithelium plays an active role in promoting colitis by shaping the tissue cytokine profile
Source: PLoS Biol. 2018 Mar 29;16(3):e2002417. doi: 10.1371/journal.pbio.2002417 (PMC5892915; doi:10.1371/journal.pbio.2002417)
Supplement: S11 Fig — (A) Microbial composition plots showing distribution of phyla and genera from mice before and after treatment with rapamycin. Each pair of columns represents the composition of fecal pellets taken from the same mouse before and after treatment. (B) Heat map of Bray-Curtis dissimilarity scores from all samples. (C) Principle coordinate analysis (PCoA) plot created using the matrix of paired-wise distance between samples calculated by the Bray-Curtis dissimilarity. Each dot on the figure represents the whole microbial composition profile. Samples are color-coded based on treatment group. Underlying numerical values are provided in S1 Data. (PDF) [file pbio.2002417.s012.pdf]

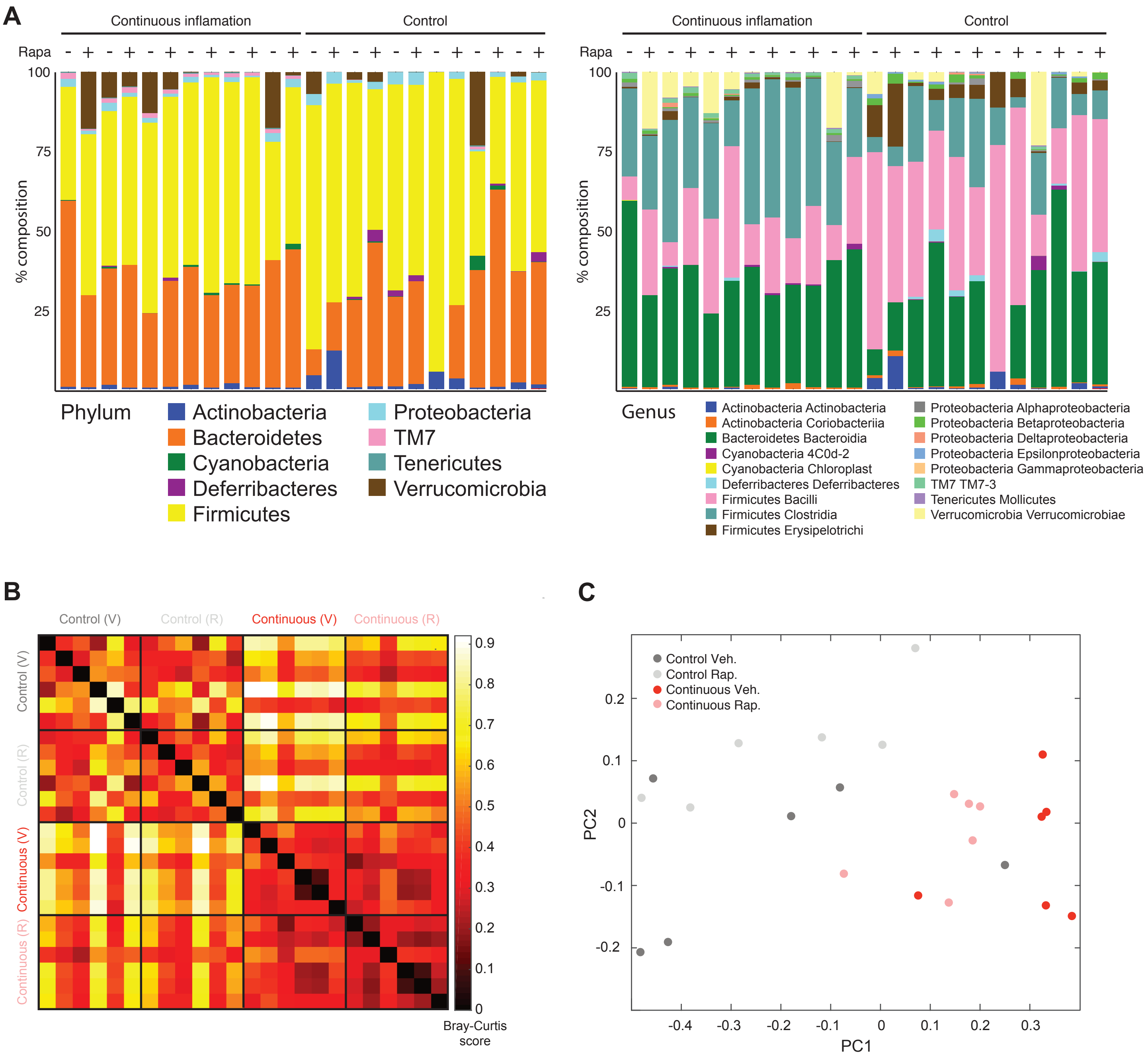

S11 Fig. Microbiome analysis of animals with inflammation. (A) Microbial composition plots showing distribution of phyla and genera from mice before and after treatment with rapamycin. Each pair of columns represents the composition of fecal pellets taken from the same mouse before and after treatment. (B) Heat map of Bray-Curtis dissimilarity scores from all samples. (C) Principle coordinate analysis (PCoA) plot created using the matrix of paired-wise distance between samples calculated by the Bray-Curtis dissimilarity. Each dot on the figure represents the whole microbial composition profile. Samples are color-coded based on treatment group.

Underlying numerical values are provided in S1 Data.
