# Supplementary material for: The colonic epithelium plays an active role in promoting colitis by shaping the tissue cytokine profile
Source: PLoS Biol. 2018 Mar 29;16(3):e2002417. doi: 10.1371/journal.pbio.2002417 (PMC5892915; doi:10.1371/journal.pbio.2002417)
Supplement: S13 Fig — Individual measurements for each cytokine, chemokine, and growth factor represented in Fig 5C. Plots represent mean +/− standard error. Significance was determined by ANOVA with Tukey-Kramer post-test. * represents p ≤ 0.05; ** represents p ≤ 0.01; *** represents p ≤ 0.001. Underlying numerical values are provided in S1 Data. (PDF) [file pbio.2002417.s014.pdf]

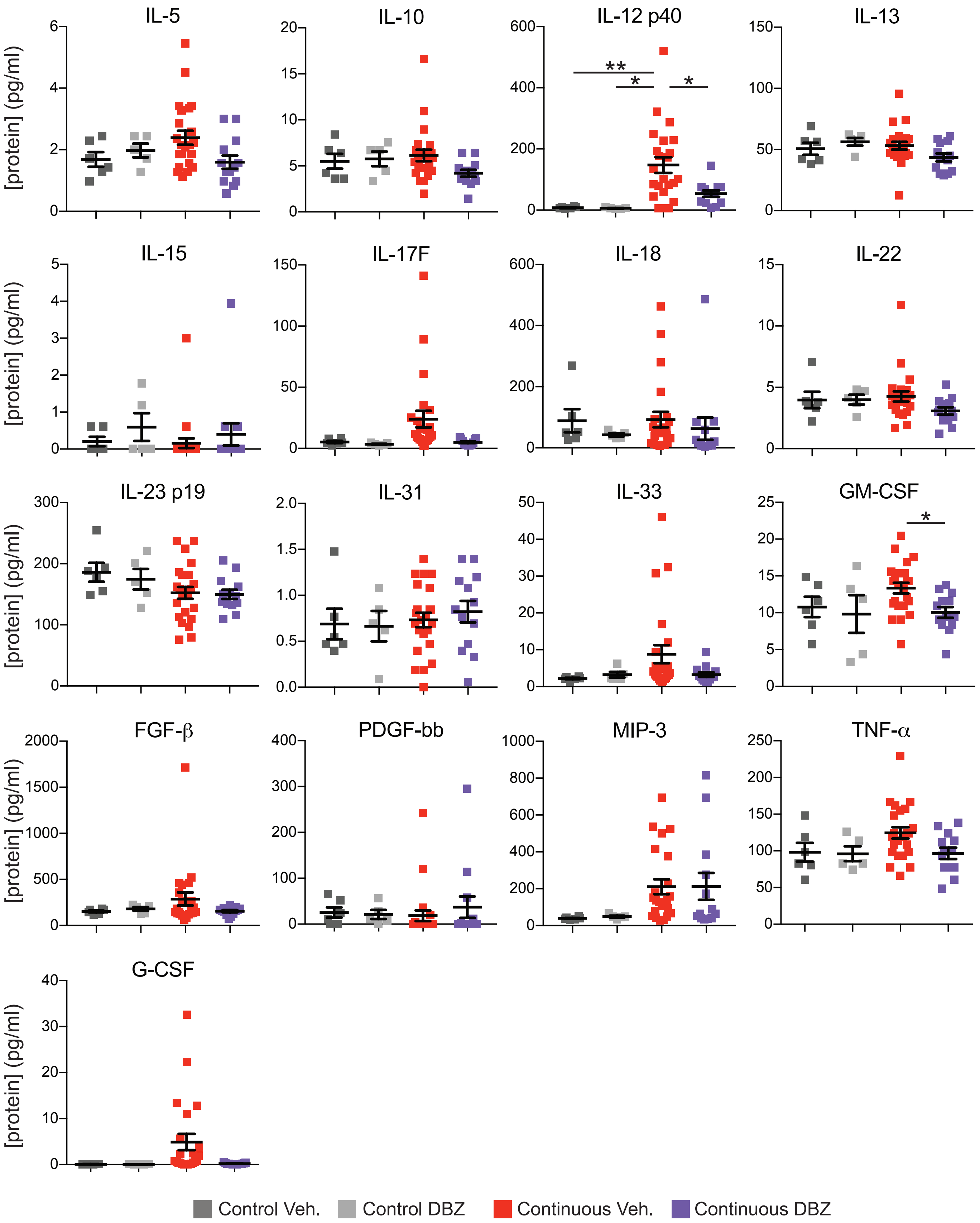

S13 Fig. Expression of cytokines, chemokines, and growth factors following dibenzazepine (DBZ) treatment. Individual measurements for each cytokine, chemokine, and growth factor represented in Fig 5C. Plots represent mean +/-standard error. Significance was determined by ANOVA with Tukey-Kramer post test. * represents $p \leq 0.05$; ** represents $p \leq 0.01$; *** represents $p \leq 0.001$. Underlying numerical values are provided in S1 Data.
