# Supplementary material for: The colonic epithelium plays an active role in promoting colitis by shaping the tissue cytokine profile
Source: PLoS Biol. 2018 Mar 29;16(3):e2002417. doi: 10.1371/journal.pbio.2002417 (PMC5892915; doi:10.1371/journal.pbio.2002417)
Supplement: S14 Fig — Treating established organoids with various drug combinations enriches for stem cells, enteroctyes, or goblet cells. Wnt-conditioned media contain EGF, Noggin, and R-spondin as well supernatants from a Wnt3A overexpressing cell line. CHIR99021 is a GSK3 inhibitor, and valproic acid (VPA) is a histone deacetylase inhibitor and Notch pathway activator. IWP-2 is a Wnt inhibitor, and DAPT is a γ-secretase inhibitor that blocks Notch pathway activity (see Materials and methods). (PDF) [file pbio.2002417.s015.pdf]

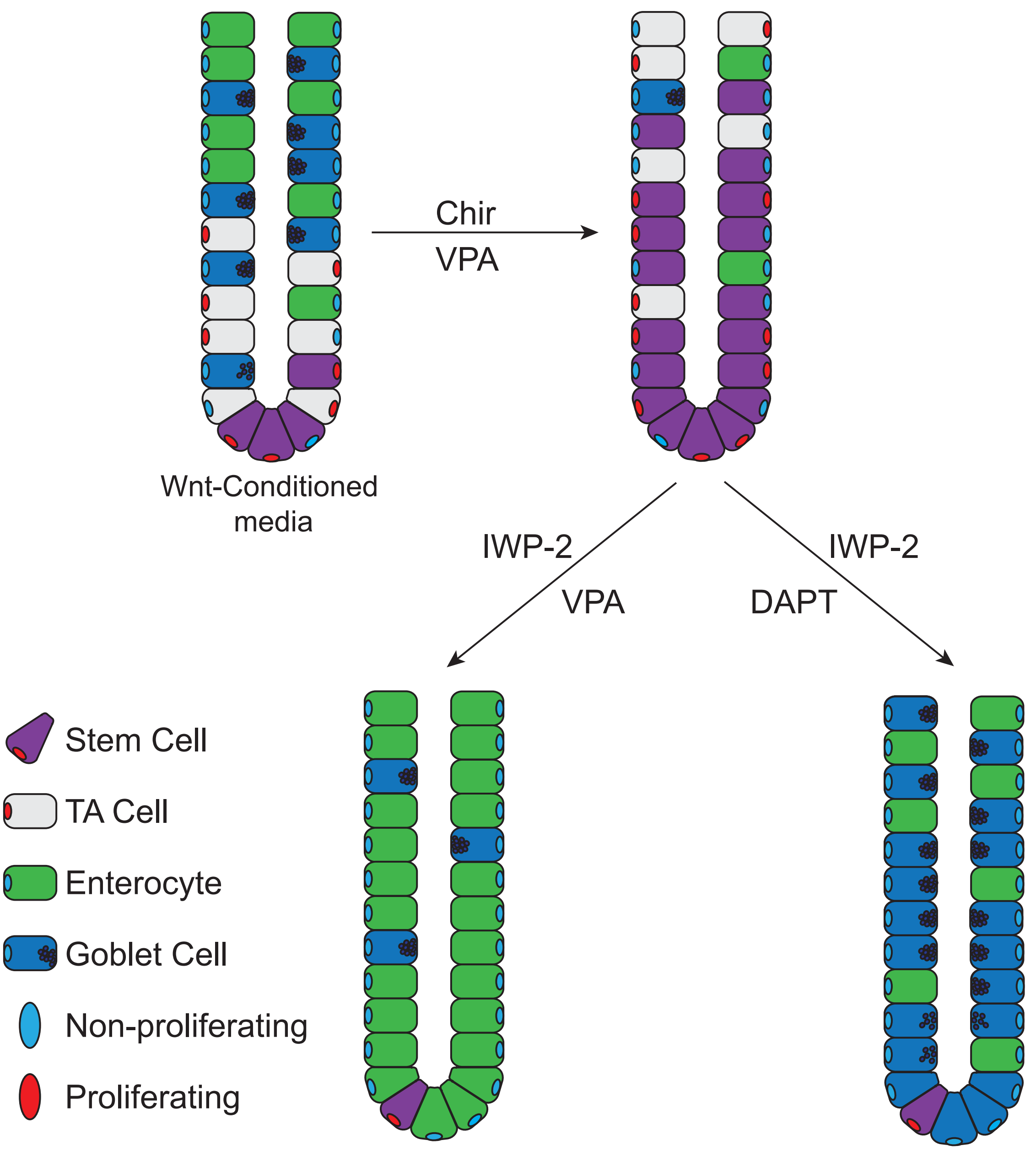


**S14 Fig. Organoid culture conditions for enriching various cell populations.** Treating established organoids with various drug combinations enriches for stem cells, enteroctyes or goblet cells. Wnt-conditioned media contain EGF, Noggin, and R-spondin as well supernatants from a Wnt3A overexpressing cell line. CHIR99021 is a GSK3 inhibitor, and valproic acid (VPA) is a histone deacetylase inhibitor and Notch pathway activator. IWP-2 is a Wnt inhibitor, and DAPT is a γ-secretase inhibitor that blocks Notch pathway activity (see Materials and methods).
